# Synergism between the Black Queen effect and the proteomic constraint on genome size reduction in the photosynthetic picoeukaryotes

**DOI:** 10.1038/s41598-020-65476-1

**Published:** 2020-06-02

**Authors:** D. Derilus, M. Z. Rahman, F. Pinero, S. E. Massey

**Affiliations:** 1 Environmental Sciences Department, University of Puerto Rico - Rio Piedras, San Juan, Puerto Rico; 2 Biology Department, University of Puerto Rico - Rio Piedras, San Juan, Puerto Rico; 3 Mathematics Department, University of Puerto Rico - Ponce, Ponce, Puerto Rico

**Keywords:** Computational biology and bioinformatics, Ocean sciences

## Abstract

The photosynthetic picoeukaryotes (PPEs) comprise a rare example of free-living eukaryotes that have undergone genome reduction. Here, we examine a duality in the process; the proposed driver of genome reduction (the Black Queen hypothesis, BQH), and the resultant impact of genome information loss (the Proteomic Constraint hypothesis, PCH). The BQH predicts that some metabolites may be shared in the open ocean, thus driving loss of redundant metabolic pathways in individual genomes. In contrast, the PCH predicts that as the information content of a genome is reduced, the total mutation load is also reduced, leading to loss of DNA repair genes due to the resulting reduction in selective constraint. Consistent with the BQH, we observe that biosynthetic pathways involved with soluble metabolites such as amino acids and carotenoids are preferentially lost from the PPEs, in contrast to biosynthetic pathways involved with insoluble metabolites, such as lipids, which are retained. Consistent with the PCH, a correlation between proteome size and the number of DNA repair genes, and numerous other informational categories, is observed. While elevated mutation rates resulting from the loss of DNA repair genes have been linked to reduced effective population sizes in intracellular bacteria, this remains to be established. This study shows that in microbial species with large population sizes, an underlying factor in modulating their DNA repair capacity appears to be information content.

## Introduction

Photosynthetic picoeukaryotes (PPEs) are single celled eukaryotic algae of cell size less than 3 µm in diameter^1^. Their individual cell size is much reduced compared to other eukaryotes, and comparable to *Prochlorococcus* and *Synechococcus* picocyanobacteria, which have also undergone cell size reduction (cell size <2 µm^2^,). They are typically motile and found in the oceanic euphotic zone^3,4^. Molecular^5^ and metagenomic^6–9^ analyses show that PPEs possess a global distribution. While factors which affect the distribution of PPEs are not well understood^3^, temperature and dissolved oxygen appear to have a role^8^. There is evidence of the importance of PPEs in biogeochemical processes such as carbon fixation^6,10^, despite their low numerical abundance compared to cyanobacteria^10^.

The reduction in cell size of both marine PPEs and picocyanobacteria has occurred concomitantly with a reduction in genome size. These two groups of photosynthetic microbes represent rare examples of free-living organisms that have undergone reduction in genome size, however the evolutionary forces and environmental factors leading to genome reduction in these two lineages are not well understood^11,12^. While these forces may be similar given their common habitat, it is unclear if they represent a case of convergent evolution, until the imperatives behind cell and genome size reduction have been determined in both groups. One potential explanation is that the cell surface/volume ratio is increased upon reduction in cell size, which enhances nutrient uptake^13^. This is consistent with the high proportion of membrane transporters in SAR11 picocyanobacterial genomes^14^.

Alternatively, the Black Queen Hypothesis (BQH) proposes that genome reduction in the picocyanobacteria is driven by the evolution of dependencies between microbes, with some gene products, and their metabolites, leaking into the aquatic medium and acting as public goods^11^. Public goods are defined as publicly available resources that are non-excludable and non-rivalous. This means that individuals cannot be excluded from using them, and use by one individual does not reduce availability to others, respectively. Presumably metabolites and gene products present in ocean water are non-rivalous, given that they are unlikely to be depleted due to the volume in which they reside, which implies they are not susceptible to over-exploitation by cheaters. The BQH proposes that the presence of such compounds in the aquatic medium may facilitate adaptive gene loss from some microbial lineages, as they no longer to synthesize the compound if they are able to scavenge it.

The BQH proposes that in the picocyanobacteria such compounds include catalase-peroxidase cross-protection, fixed nitrogen, iron carrying siderophores and reduced sulfur (dimethylsulfoniopropionate)^11^. The concentration of the compound in the aquatic medium needs to be high enough so that a microbe that has undergone gene loss may be able to effectively use it; this appears true of reduced sulfur^15^, iron carrying siderophores^16^ and fixed nitrogen in the form of ammonia^17^. Specialized membrane transporters may help to address the problem of those compounds that are present in the aquatic medium at low concentration. Particularly, specific siderophores require specific uptake transporters, and this means that they only appear to act as a public good amongst strains of the same genotype^18^. In the case of PPEs, a fundamental prediction consistent with the BQH is that genes for the biosynthesis of soluble metabolites should be preferentially lost, as opposed to those for insoluble metabolites, which cannot be easily shared in an aqueous medium. In addition, such soluble metabolites should be present at sufficient concentration in the open ocean that they can compensate for gene loss.

Sanctioning promotes cooperation in public goods games^19^, and so it may be proposed that forms of sanctioning at the microbial level may help to promote the fair use of microbial public goods. This can include Hamiltonian spite^20^, a sanctioning behavior that has a negative impact on both actor and recipient, who are unrelated^21,22^. Game theoretic approaches inspired by economics have been brought to bear on the freeloading problem of microbial public goods^23^, and contract theory has also been explored in the context of organismal mutualism^24–26^. However, adhering to the strict definition of public goods that they are non-rivalrous means they are not susceptible to freeloading/cheating behaviors, as they cannot be over-exploited. This is likely the case with metabolites and other gene products present in the open ocean.

The Proteomic Constraint Hypothesis (PCH) proposes that a secondary effect of a reduction in genome size is the concomitant reduction in the selective constraint that maintains genes involved in informational fidelity^27^. This is because an effect of genome reduction is to reduce the amount of coding information, and so the size of the mutational target. This means that the overall mutational load will decrease, thus any primary genome reduction would be expected to loosen the selective constraint on DNA repair genes, and other genes involved in maintaining informational fidelity, leading to their secondary loss. This leads to the prediction that the numbers of DNA repair genes, which reflects the DNA repair capacity, should correlate with proteome size. Such a correlation is observed in bacteria^28,29^, archaea and DNA viruses^30^, but has not yet been examined in eukaryotes.

Lastly, intracellular bacteria have undergone a reduction in genome size, which has been linked to a reduction in population size and a resulting increase in genetic drift. Gene loss in these lineages is postulated to result from the reduction in selection pressure that results from an increase in genetic drift^31^, however this remains to be established. Presumably, enhanced drift is not a factor in gene loss in the PPEs, which are likely to have large population sizes due to their oceanic distribution, and for which there is some genomic evidence^32^. In this work, we test the potential effects of these three different scenarios on genome size reduction, using a comparison of 16 unicellular eukaryotic algal genomes.

## Methods

### Collection of proteome and genome sequences

Genome and proteome sequence data for all 16 unicellular photosynthetic algae available at the start of the analysis were downloaded from the National Center for Biotechnology Information (NCBI) (July 2018). *N.gaditana* has a low number of genes, it remains to be established if this is due to incomplete gene identification and annotation. The selected species were divided in two groups based on their genome and cell size: 1) the photosynthetic picoeukaryotes (PPEs) with cell size lower than 3 µm (n = 7), and 2) the photosynthetic micro-algae (PMA) with cell size higher than 3 µm (n = 9). PPE genome size varies between 12 Mb (*Ostreococcus tauri)* to 15 Mb (*Bathycoccus prasinos*), and PMA genome size varies from 23 Mb (*Auxenochlorella protothecoides*) to 137 Mb (*Volvox carteri*). The standard used to select an organism for this survey required the complete genome annotation and publicly available experimental references. The key genomic and proteomic features of the 16 unicellular photosynthetic algae species used for the analysis are supplied in Table 1.

**Table 1:** Key genomic and physical features as well as the assembly accession number of the unicellular algal species included in the comparative genomic analysis.

Species	Genome size (Mb)	Minimum size (µm)	Maximum size (µm)	Category	Reference	NCBI assembly accession number
*Auxenochlorella protothecoides*	23	4	8	PMA	^76^	GCA_000733215.1
*Bathycoccus prasinos*	15	1.5	2.5	PPE	^77^	GCA_002220235.1
*Chlamydomonas reinhardtii*	120	NF	10	PMA	^78^	GCA_000002595.2
*Chlorella variabilis*	46	2	10	PMA	^79^	GCA_000147415.1
*Chrysochromulina sp CCMP291*	59	NF	4	PMA	^80^	GCA_001275005.1
*Coccomyxa subellipsoidea*	49	3	9	PMA	^81^	GCA_000258705.1
*Micromonas commoda*	21	1	2	PPE	^82^	GCA_000090985.2
*Micromonas pusilla*	22	1	3	PPE	^3^	GCA_000151265.1
*Monoraphidium neglectum*	70	10	20	PMA	^83^	GCA_000611645.1
*Nannochloropsis gaditana*	34	2	4	PPE	^3^	GCA_000240725.1
*Ostreococcus lucimarinus*	13	0.8	1.1	PPE	^3^	GCA_000092065.1
*Ostreococcus sp RCC809*	13	0.8	1.1	PPE	^3^	OstRCC809_2*
*Ostreococcus tauri*	12	0.8	1.1	PPE	^3,84^	GCA_000214015.2
*Thalassiosira oceanica*	92	3	12	PMA	^3^	GCA_000296195.2
*Thalassiosira pseudonana*	32	2.3	5.5	PMA	^3^	GCA_000149405.2
*Volvox carteri*	137	FD	500	PMA	^85^	GCA_000143455.1

All species with average cell size less than 3 µm were grouped as PPE (photosynthetic picoeukaryote) and those with cell size higher than 3 µm were grouped as PMA (photosynthetic microalga).

*JGI identifier, NF: minimum cell size not found in the literature.

### Orthogroup inference

An orthogroup (OG) is a set of genes that descended from a single ancestral gene for a group of species. Hence an orthogroup, which contains both orthologs and paralogs, is considered a basic unit for this comparative genomics survey, and due to common ancestry consists of genes of related function. Orthofinder version 2.2.6^33^ was used to identify OGs in the 16 genomes. OGs were inferred by the following Orthofinder command line: /orthofinder -f proteomes/ -M msa. The -M msa parameter was used to infer maximum likelihood trees from multiple sequence alignment (MSA) methods. The OG inference produces a set of files describing orthologs, paralogs, OGs, gene trees, ortholog alignment, gene duplication events, and additional comparative genomics statistics for all the species analyzed.

### Functional annotation of the orthogroups

We developed a multi-species approach to functionally annotate the identified OGs. This approach involves two steps: (i) KEGG Orthology (KO) annotation of each genome separately, followed by (ii) KO mapping of the annotated genes to the entire OGs for all 16 species.

#### KEGG Orthology (KO) annotation

The KEGG Orthology (KO) assignment was conducted for each individual genome separately. This was done by a Blastp search of the protein *fasta file for each species against the non-redundant protein NCBI database, with an e-value cut-off of 1e^−10^. From this blast output, the gene ID and Genbank ID numbers (GI) were retrieved and sorted. The resulting GI numbers were converted to UniProt and then to K numbers subsequently using an in-house ID mapping python script that can be obtained from github.com/dieunelderilus/picoeukaryotes/blob/master/gi_kO_mapper.py. Briefly, this script takes as input a table with gene ID and GI numbers for the considered species and outputs a comma separated table which links each individual gene ID to its corresponding UniProt and K number respectively (GeneID→GI→UniProt ID→K).

#### Mapping of KO annotation to orthogroups

The unicellular eukaryotic algae are not well annotated in KEGG. To improve the annotation, the file linking Gene ID to K number for each individual species was used to perform a KO assignment to the orthologous genes found in the ‘Orthogroups.tsv’ file generated by the Orthofinder analysis. The ‘Orthogroups.tsv’ is a tab separated file that displays the OGs identified. The idea of our ID mapping strategy is that any K number assignment for one gene ID of a set of homologous genes (from the same orthogroup), could be extended to all the genes in this OG (for the 16 species) that failed to be annotated in the first round of ID mapping.

After successful KO assignment of the OGs for all 16 species, the most common K number was selected and assigned to all the homologous genes that belonged to the considered OG. This approach improves significantly the functional mapping efficiency, which is a common problem in functional comparative genomic studies. The efficiency of the single species Method 1 (M1) compared to our multiple species annotation Method 2 (M2) is shown in Supplementary Fig. 1, where we show that M2 significantly improves ID mapping efficiency. The resulting functional orthologs found for different species were quantified by mapping them against the OG inventory found in ‘Orthogroups.GeneCount.csv (orthofinder output) file, which contains the number of genes in each OG for each species.

### Mapping K numbers to KEGG pathways

After assigning K numbers to individual genes and OGs for all the genomes, the relative abundance of genes in different categories of metabolic pathway were determined. These were mapped against the KEGG pathways database which was downloaded from www.kegg.jp (Last updated: August 21, 2018) and reformatted with an in-house python script. The ID mapping output constitutes a key piece of data facilitating study of the metabolic diversity found within the single-celled algal genomes, specifically with regard to genome size reduction, and concomitant changes in metabolic functionality.

### Phylogenomic analysis

In order to determine the phylogenetic relationship of the 16 species, phylogenomic analysis was conducted using 548 core orthogroups, which are the set of OGs containing at least one gene copy from each of the 16 genomes analyzed. Our gene clustering analysis pipeline generated a Multiple Sequence Alignment (MSA) for each individual OG. The resulting MSA for the core OGs was processed as follows: (1) duplicate sequences were removed in each individual MSA; (2) poorly aligned regions were removed using the –gappyout option of trimAl v1.4^34^. The trimming option selects the best threshold, based on the combination of gap and similarity scores; (3) the number of reads for each MSA was confirmed to be 16, which corresponds to the number of species and (4) all MSAs comprising the core proteome were concatenated using AMAS^35^.

This produced a nexus file containing 208426 amino acid sites, 184449 (88%) variables sites, 150691 (72%) parsimony informative sites and 3334816 matrix cells with 517089 (15%) undetermined characters. The initial alignment was further trimmed using Gblocks (Version 0.91b)^36^ with stringent selection parameters. This final filtering step resulted in a concatenated alignment containing 28713 amino acid sites, 23243 (81%) variables sites, 18843 (66%) parsimony informative sites and 4549408 matrix cells with 25 (0.005%) undetermined characters. This final alignment was used for the phylogenomic construction of the PPEs and PMAs.

### Construction of the phylogeny

Using Modelfinder^37^ according to the Bayesian Information Criterion (BIC), the most appropriate protein substitution model was identified as LG + F + R5^38^. Markov Chain Monte Carlo (MCMC) simulation was performed in MrBayes v3.2.5^39^ for the phylogenomic analysis. The analysis was conducted for a total of 300000 generations and a sample frequency of 30. These parameter values ensure that at least one hundred thousand (100000) samples formed the posterior probability distribution. Next, a consensus tree was generated after discarding 25% (2500) of the initial run as burn in, producing a cladogram with the posterior probabilities for each split and a phylogram with mean branch lengths.

To test the consistency of the tree topology, maximum likelihood (ML) analysis was performed in RaxML v 8.2.12^40^. First, 20 ML trees were generated using the command line raxmlHPC -m PROTGAMMALG -p 12345 -# 20 -s concatenated_prototein.py -n T1, and the tree with the best likelihood saved to a file called RAxML_bestTree_T1. Secondly, to obtain support values a bootstrapping (n = 100 replicates) was performed using the command raxmlHPC -m PROTGAMMALG -p 12345 -b 12345 -# 100 -s concatenated_prototein.py -n T2, which prints bootstrap replicate trees to RAxML bootstrap.T2. Thirdly and finally, the ML best-fit and the bootstrapped trees were used to generate the bipartition trees with the following command: raxmlHPC -m PROTGAMMALG -p 12345 -f b -t RAxML_bestTree.T13 -z RAxML bootstrap. T14 -n T3. This last step generated a bipartition tree (with support values assigned to branch and nodes), which was displayed in Mega7^41^, for comparison with the tree generated by MrBayes^39^. The tree was used for phylogenetic independent contrasts (PIC) correction^42^ of correlations between proteome and genome size, and differing categories of orthgroups, based on their K numbers.

### Network tree construction

Metabolic networks were generated for each of the 16 genomes, as follows. The annotated genes of each individual genome were assigned to KEGG Orthology (KO) as described above. This resulted in a list of K numbers for each species which were converted into reaction numbers (rn). Subsequently, the rn numbers were converted into an edge list linking two or more compounds with biological functions (cpd) via a mapping file (containing KEGG objects that are associated with genes, proteins, small molecules, reactions, pathways, diseases and drugs) obtained from the KEGG database (www.genome.jp/kegg/). The metabolic network for each individual genome was visualized in Gephi^43^, and the relative abundance of enzymes responsible for different reactions is reflected in the edge width between cpd nodes. The following network similarity indices were used:

*Unweighted Jaccard Index*. If *I* and *J* are sets then the unweighted Jaccard index of the similarity between *I* and *J* is

$$Jac(I,\,J)=\frac{|I{\cup }^{}J|-|I{\cap }^{}J|}{|I{\cup }^{}J|}=1-\frac{|I{\cap }^{}J|}{|I{\cup }^{}J|}$$

where $|I{\cap }^{}J|$ is the number of elements common to both *I* and *J* and $|I{\cap }^{}J|$ is the number of elements in either *I* or *J*. However, this similarity index considers only the presence or absence of a particular factor. It does not consider the strength or magnitude of any of the factors. The weighted Jaccard Index takes into account the magnitude.

*Weighted Jaccard Index*. If *x* and *y* are vectors of real numbers of the same length, the weighted Jaccard index of the similarity between *x* and *y* is

$$WJac(x,y)=\frac{{\sum }^{}(\max ({x}_{i},\,{y}_{i}))-{\sum }^{}(\min ({x}_{i},\,{y}_{i}))\,}{{\sum }^{}(\max ({x}_{i},\,{y}_{i}))}=1-\frac{{\sum }^{}(\min ({x}_{i},\,{y}_{i}))}{{\sum }^{}(\max ({x}_{i},\,{y}_{i}))}$$

*Canberra Distance*. If *x* and *y* are vectors of real numbers of the same length, the Canberra distance between *x* and *y* is

$$Can(x,y)={\sum }^{}\frac{|{x}_{i}-{y}_{i}|}{|{x}_{i}|+|{y}_{i}|}$$

If *NZ* is the number of nonzero positions in both *x* and *y* then the Adkins form of the Canberra distance is

$$AdCan(x,y)=\frac{1}{NZ}{\sum }^{}\frac{|{x}_{i}-{y}_{i}|}{|{x}_{i}|+|{y}_{i}|}$$

Note that as $|{x}_{i}-{y}_{i}|=\,\max ({x}_{i},{y}_{i})-\,{\rm{\min }}({x}_{i},{y}_{i})$ we may rewrite the Canberra distance as

$$Can(x,y)={\sum }^{}\frac{\max ({x}_{i},{y}_{i})-\,{\rm{\min }}({x}_{i},{y}_{i})\,}{|{x}_{i}|+|{y}_{i}|}\,{\rm{and}}\,AdCan(x,y)=\frac{1}{NZ}{\sum }^{}\frac{\max ({x}_{i},{y}_{i})-\,{\rm{\min }}({x}_{i},{y}_{i})\,}{|{x}_{i}|+|{y}_{i}|}$$

The biggest difference between the weighted Jaccard index and the Canberra distance is that the Canberra distance computes the similarity of terms *x*_*i*_ and *y*_*i*_ first and then sums the overall similarities, whereas the weighted Jaccard index finds the number of common elements to both *x* and *y*, and the number of elements in either *x* or *y* first and division is performed last. An example illustrating the differences between the distance measures is provided in Supplementary Material. Distances generated using the above measures were used as input into Phylip^44^ to construct a tree of metabolic networks, using the neighbor joining method^45^. Congruence with the phylogenomic topology was calculated using comparePhylo {ape} R package^46^.

### Population size estimation using MSMC

Genome wide SNPs were generated for *O.tauri* strain RCC1116 using the reference genome sequence (Genbank Assembly ID GCA_000214015.2) and raw sequencing reads (accession number SRR4026808) obtained from the NCBI. The complete pipeline used to generate the SNPs from the raw reads can be found at github.com/dieunelderilus/picoeukaryotes/blob/master/SNP_calling.sh. Briefly, the raw reads were filtered using fastp^47^, then mapped to the reference genome using BBMap (sourceforge.net/projects/bbmap/). The resulting sam files were converted to bam, and sorted using samtools^48^, before duplicate removal using Picard (broadinstitute.github.io/picard/), and indel realignment using GATK^49^. SNP calling was conducted using bcftools mpileup^48^, with filtration parameters of Q ≥ 20 and depth of coverage (DP) ≥ 5. The SNP calling procedure generates a similar number of SNPs (54527) to those reported in the literature for *O.tauri* strain RCC1110 (47502), using a related SNP calling procedure^50^.

MSMC2^51^ was run with the option -p 1*2+15*1+1*2, which takes into account reduced genome size. A spontaneous mutation rate of 4.80E-10 mutations per nucleotide per generation^52^ and a generation time of 11.3 hours^53^ were used to estimate the effective population size of *O.tauri*. The population size estimate was taken from the midpoint of the simulation which corresponds to 14000 generations.

## Results and Discussion

### Orthogroup analysis

**Figure 1:**
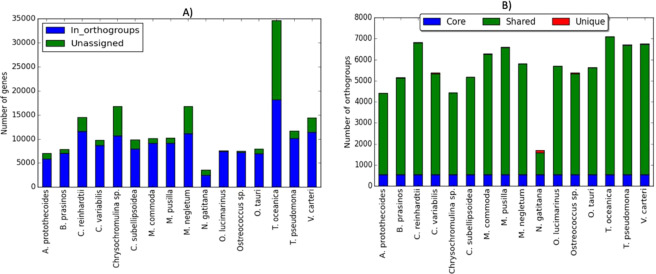
Orthogroup analysis results. Bar plot presenting (**A**) the number of genes for each species, and the number genes assigned to orthogroups (OGs). (**B**) the number of OGs for each species emphasizing the contribution the contribution of core OGs (found in all the 16 species), shared OGs (assigned to more than one but not all 16 species) and unique OGs (species specific OGs).

The OG analysis resulted in a total of 14651 OGs, distributed amongst the 16 species (Fig. 1). Out of a total of 190314 genes, 145163 (76.3%) were contained in OGs. The remaining 23.7% unassigned genes may be considered as species-specific genes. The average OG size was 10 genes, while 4004 (27%) of the total OGs consist of only two genes. The OG size ranged from 2 to 1700 genes per OG. A total of 14651 OGs was inventoried for the 16 species (the pangenome), from which the core genome (554 OGs that are found in all 16 species), shared genes (5187 OGs that are found in more than one but not in all species), and unique genes (309 OGs, considered as species-specific genes) was determined (Supplementary Table 1, Supplementary Fig. 2).

No statistical difference was found in the number of OGs between PPE and PMA genomes, despite greater gene loss from PPE genomes (Supplementary Fig. 3). This indicates that during the genome reduction of PPEs, there may have been an advantage in maintaining gene family diversity at the expense of gene family size, by the preferential loss of paralogs, consistent with^54^. The Black Queen effect and loss of redundant genetic factors may act synergistically to drive the genome reduction of PPEs.

### Phylogenomic analysis

**Figure 2:**
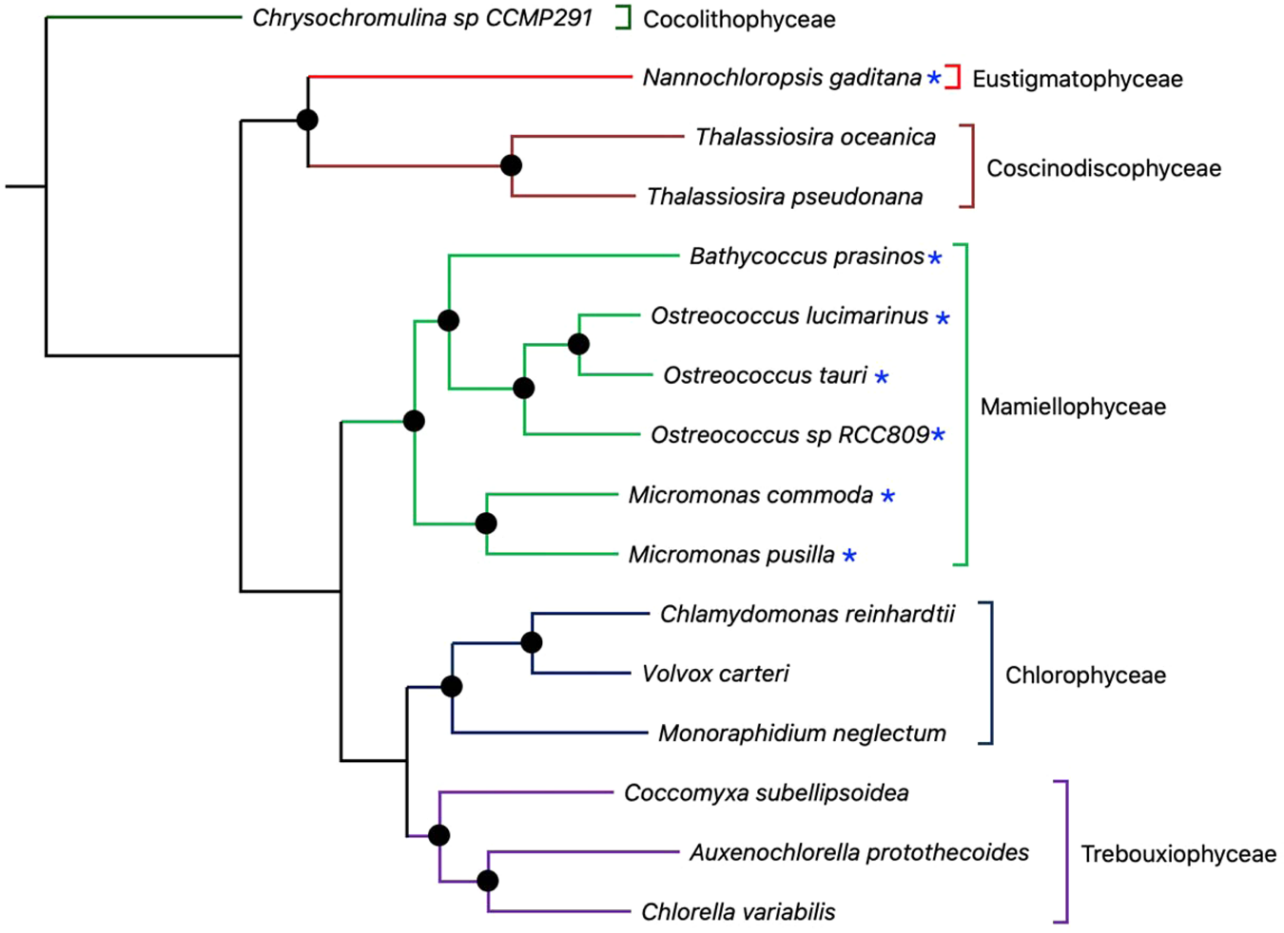
Phylogenomic tree of 16 unicellular photosynthetic algae, highlighting the phylogenetic position of the photosynthetic picoeukaryotes (PPEs). The tree was inferred using MrBayes using the core proteome (comprised of core orthogroups) of the 16 algal species. Solid black circles indicate nodes supported with posterior probabilities of 1. The tree topologies produced under Bayesian and ML (not shown) methods were identical, and all nodes received bootstrap support of 100% (ML) and posterior probabilities of 1.0 (Bayesian). Different branch colors indicate different classes which are indicated by brackets to the right. The position of the PPE species is highlighted with an asterisk. The outgroup consists of ‘*Chrysochromulina* sp CCMP29’, a member of the Coccolithophyceae.

The phylogenomic approach tends to produce a better approximation to the true species tree than when using a single gene for tree reconstruction. To determine the broader evolutionary history of PPEs and their phylogenetic relationships, we constructed Bayesian and ML phylogenies, using the core genome identified from the OG analysis (Table 1). After trimming and filtering, a final alignment was obtained of 28713 amino acid sites, which consisted of 23243 (81%) variables sites, 18843 (66%) parsimony informative sites and 4549408 matrix cells with 25 (0.005%) undetermined characters. The MrBayes analysis reached convergence after 300000 generations. We found that the topologies produced under Bayesian and ML methods were identical, and all nodes received bootstrap support of 100% (ML) and posterior probabilities of 1.0 (Bayesian) (Fig. 2).

From our phylogenomic analysis two major clades were identified: 1) a strongly supported clade of 12 species and distributed through three main classes (Trebouxiophyceae, Chlorophyceae and Mamiellophyceae) and one phylum (Chlorophyta); 2) a second clade of 3 species spread over two classes (Coscinodiscophyceae and Eustigmatophyceae) and one phylum (Heterokonta). The species *Chrysochromulina* sp CCMP29 which belongs to the class Coccolithophyceae and phylum Haptophyta, was used as an outgroup to the rest of the species analyzed, consistent with its basal nature^3^.

The phylogenomic analysis reveals a sister relationship between the Trebouxiophyceae and Chlorophyceae clades, consistent with a previous chloroplast phylogenomic tree^55^. Members of the class Mamiellaceae formed a sister group with the Chlorophyceae/Trebouxiophyceae clade, in agreement with previous phylogenetic analysis based on single 18S rRNA gene sequences^3,56^ and concatenated gene sequences^57^. Within the Mamiellaceae class, *Micromonas* sp. were basal, followed by *Bathycoccus* sp., consistent with a phylogenetic analysis that used 16 concatenated plastid genes^58^. The branching pattern of *Ostreococcus* sp. is inconsistent with a phylogenetic analysis that used the rRNA operon^59^. In the latter study, the statistical support was 0.88 (posterior probability), while in our study the branching pattern is strongly supported. Taken together, the phylogenomic analysis supports polyphyly of the PPEs between the Mamiellophyceae and Eustigmatophyceae. The polyphyly of the PPEs suggests that genome reduction occurred more than once independently and can be observed in Fig. 2.

### Network tree analysis

Network tree analysis is a new approach for examining relationships between empirical networks^4,60,61^. In the context of this study, the approach may help to reveal the dynamics of pathway loss during genome reduction. We generated a cpd list (compounds with biological function) for each individual species. After redundancy removal, the number of cpd nodes shared between each pair of networks was calculated (Supplementary Table 2). Sampling bias is minimized due to our sequence based OG identification approach, which relies on the accurate determination of protein coding gene presence/absence from all of the 16 genomes. Protein coding gene identification approaches are typically accurate, and so the identified proteins from each genome should be comparable.

A distance matrix was generated using our previously described network alignment approach that utilizes the Jaccard Similarity Index^61^ and used as input to generate a neighbour joining tree. The overall topology of the metabolic network tree is largely inconsistent with the topology of the phylogenomic tree, showing only 6% shared nodes (Supplementary Fig. 5A). When the weighted Jaccard Similarity Index was used for tree construction (Supplementary Fig. 5B), an improvement was observed of 25% shared nodes with the phylogenomic topology.

**Figure 3:**
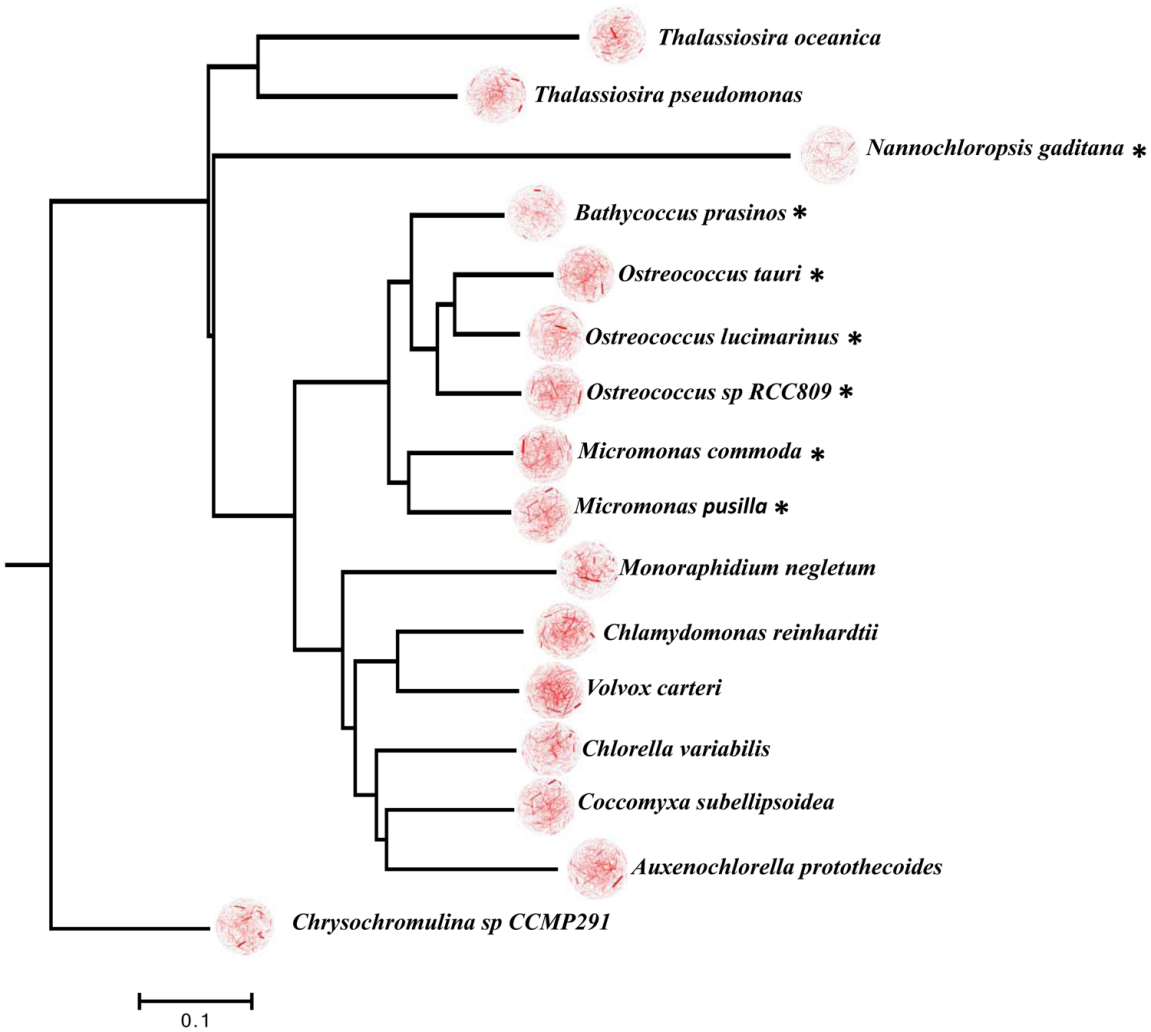
Neighbor joining tree of 16 genomic metabolic networks belonging to the PPEs. The tree was generated in Phylip with a lower-triangular data matrix which contained the Adkins Canberra distance between each pair of networks. 3D meta-metabolic networks are shown for each species, to the left of the species name, and the PPE species are highlighted with an asterisk.

Near congruence is observed between the network tree generated using the Adkins Canberra distance (Fig. 3) and the phylogenomic tree (80% shared nodes), despite the low annotation of the picoeukaryotes genomes analysed in KEGG database. On the network tree, the position of *Nannochloropsis gaditana* is basal to the chlorophytic algae, which is incongruent with the phylogenomic tree. On the network tree the *N.gaditana* displays a long branch, resulting from its extreme genome reduction, which helps to explain its incongruence with the species tree. The position of *Monoraphidium neglectum* (Chlorophyceae) is also incongruent with the phylogenomic tree, being basal to both Chlorophyceae and Trebouxiophyceae in the network tree. The reason for this is unclear but indicates a significant metabolic deviation from the other members of the Chlorophyceae and Trebouxiophyceae.

The higher overall level of congruence observed when the Adkins Canberra distance is used indicates that it is a superior measure of network similarity. The near congruence with the phylogenomic tree implies that the genome-scale metabolic networks are influenced by phylogenetic inertia, reflecting the influence of gene gain and loss events in common ancestral lineages. The position of lineages with reduced genome and proteome sizes on the metabolic network tree are displayed in Supplementary Fig. 5.

The placement of *N.gaditana* on the metabolic network tree separate from the other PPEs, which form a single clade, indicates that it represents a distinct ecophysiotype, and that metabolically it is not convergent with PPEs that belong to the Mamiellophyceae. Thus, the analysis reveals at least two distinct ecophysiotypes within the PPEs, and additional ecophysiotypes may be characterized when additional PPE genomes are sequenced. It is likely that PPEs from distinct phylogenetic lineages represent distinct ecophysiotypes. While taxa from these lineages will likely have undergone genome reduction, the analysis implies that functional metabolic convergence may not be observed between different PPE groups.

### Correlation between proteome size and metabolic capacity, and other cellular functions

Genes annotated in the genomes of the 16 unicellular chlorophytic algae belonged to 249 KEGG functional categories, of which 83 (33%) exhibit significant positive correlations with proteome size after PIC correction (R > 0.5, p < 0.0.05) (Supplementary Table 2). Interestingly, several of these categories are involved in the production of soluble metabolites, these include arginine biosynthesis (KO00220), lysine biosynthesis (KO00300), phenylalanine, tyrosine and tryptophan biosynthesis (KO00400), valine, leucine and isoleucine biosynthesis (KO0290), carotenoid biosynthesis (KO00906), ubiquinone biosynthesis (KO00130) and monoterpenoid biosynthesis (KO00902).

**Figure 4:**
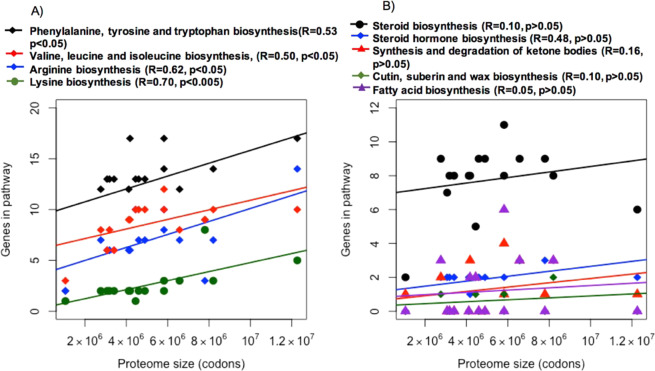
Correlation between the number of genes involved in amino acid (**A**) and lipid (**B**) biosynthesis pathways, with proteome size. After PIC correction, a significant positive correlation was observed between proteome size and the number of functional genes involved in amino acid biosynthesis, which are considered as water soluble products. However, no significant correlations were observed between proteome size and genes involved in biosynthesis of lipids, which are insoluble.

However, functional categories related to the biosynthesis of water-insoluble metabolites were not significantly correlated with proteome size (Supplementary Table 3). These include fatty acid biosynthesis (KO00061), lipid biosynthesis (KO01004), lipopolysaccharide biosynthesis (KO01005), glycosphingolipid biosynthesis (KO00603), steroid biosynthesis (KO00100), and cutin, suberin and wax biosynthesis (KO00073). The correlations between the number of genes involved in amino acid (water-soluble) and lipid (water-insoluble) biosynthesis with proteome size are shown in Fig. 4A,B, respectively (in Fig. 4B, the species with the largest proteome size (*T.oceanica*) has an unusually low number of genes in the differing categories, which may have the effect of reducing the gradient of the slope. Due to the limited number of genomes available from unicellular algae, it is unclear if *T.oceanica* is representative of its taxonomic group in terms of gene numbers). These observations are consistent with the BQH, which implies that insoluble metabolites should be retained, as they cannot be shared in the aquatic medium. In microalgae, a conspicuous nutritional deficiency in numerous taxa is that of^62^, however the corresponding KEGG categories (KO00730 and KO00780, respectively) did not show a relationship with proteome size.

**Figure 5:**
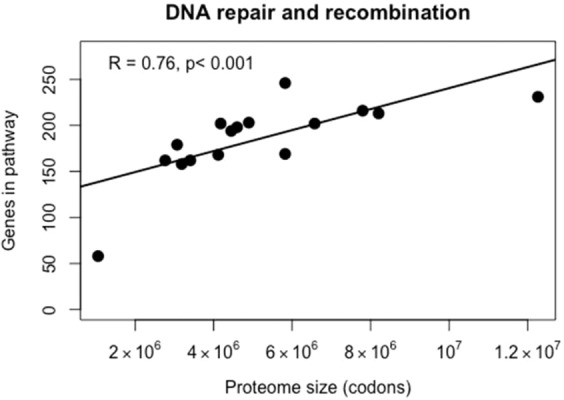
Correlation of the number of genes involved in DNA repair and recombination with proteome size, after PIC correction.

A strong positive correlation (R = 0.76, p < 0.001) was observed between proteome size and DNA repair pathways (KO03400, Fig. 5), indicating that genes for DNA repair are lost as genome size reduces. This might be expected to lead to an accelerated evolutionary rate, reflected in elongated branch lengths on a phylogenomic tree. However, no correlation was found between proteome size and phylogenomic tree branch length (the branch length was measured from the ancestral node of all species, to the branch tip for each lineage, Supplementary Fig. 6). This result contrasts with qualitative observations made in lineages of intracellular bacteria and microsporidia, which have accelerated evolutionary rates associated with genome reduction, reflected in relatively long branch lengths on phylogenetic trees^63–67^. However, phylogenetic trees measure substitution rates which are influenced by both underlying mutation rate and replication rate. Since replication rate is hard to measure, this complicates attempts tto attribute accelerated evolutionary rates to loss of DNA repair genes.

**Figure 6:**
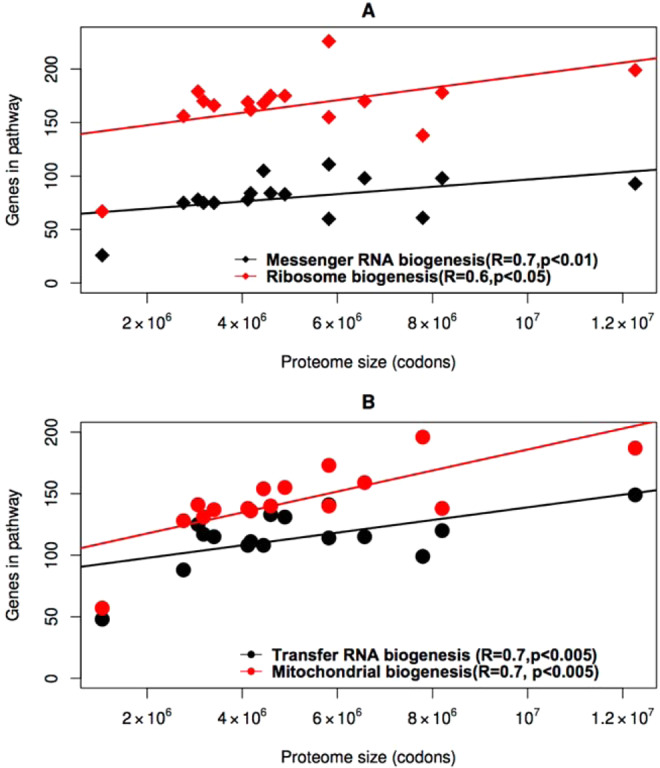
Correlation between the number of genes involved in genetic information processing and proteome size. After PIC correction, we found strong and significant positive correlation between proteome size and genes involved in mRNA (R = 0.7, p < 0.05), and ribosome (R = 0.6, p < 0.5) biogenesis (**A**). The number of functional genes associated with mitochondrial and transfer RNA biogenesis was also significantly correlated to proteome size (R = 0.7, p < 0.05) (**B**).

With a reduction in proteome size, numerous additional categories associated with informational processes lost OGs, consistent with the predictions of the PCH^27^. These categories include translation factors (KO03012), ribosome biogenesis (KO03009), spliceosome (KO03041), transfer RNA biogenesis (KO03016), aminoacyl-tRNA biosynthesis (KO00970), chaperones and folding catalysts (KO03110) and messenger RNA biogenesis (KO03019). Strong and significant positive correlation between proteome size and the number of genes associated with genetic information processing is depicted in Fig. 6.

Additional categories that have lost OGs as proteome size has reduced include transporters (KO02000) and peroxisome proteins (KO04146). The loss of peroxisome proteins is consistent with the BQH, which explains the independent loss of the catalase-peroxidase gene (*katG*) from several lineages of cyanobacteria^11^, by proposing that catalase-peroxidase is released into the marine medium after lysis of cells that possess the enzyme, however experimental evidence for this is currently lacking. The correlation strength (R) and significance (p-value) between all biosynthesis and biogenesis pathways (n = 40), and proteome size, are supplied in Supplementary Table 4. In addition, the degree of reduction in number of genes from the largest proteome size to the smallest proteome size was calculated, using the best-fit line as reference. The number of complete genomes used in the analysis is limited. As such, some data points may have an influence on the overall slope of the best fit lines, and R values. Additional complete genome sequences will help to clarify the results reported here.

### Genome reduction, differential gene loss and the Black Queen

Despite an improvement in ID mapping efficiency due to our procedure described in Methods, the overall efficiency was 32% of all genes in the 16 genomes assigned a K number. Thus, we only assessed a proportion of the total number of genes present, for the effects of genome reduction. However, correlations within this subset are meaningful as they represent a subsample and reveal important information regarding the dynamics of genome reduction. While the subset is not random but biased towards genes that have had a greater research impetus to be annotated, an argument can be made that these genes are more likely to have functional significance in the genomes examined.

An alternative explanation to the BQH for differential gene loss, is that PPEs form tight syntrophic interactions with bacteria, which provide the nutrients corresponding to pathways lost from PPE genomes. This has been postulated as a solution to the freeloading of bacterial metabolites that are exchanged in syntrophic bacterial interactions^68^. However, microscopic evidence for such physical associations between the PPEs and syntrophic bacteria is currently lacking^69^, although there is some sequence evidence for an association^70^. According to the BQH, cell lysis constitutes the distribution mechanism of donors. Presumably, transporters would need to evolve in the recipient species to benefit from the metabolites, but these show a positive correlation with proteome size in the PPEs, and so are reduced in numbers in smaller proteomes.

Lastly, a reduced *N*_*e*_ has been posited as the cause of genome reduction in intracellular bacteria via gene loss by drift, and so this factor was examined in *O.tauri*. The MSMC analysis uses the expectation-maximization algorithm for parameter estimation; convergence was achieved at i = 100 (Supplementary Fig. 7). The analysis indicates that the *N*_*e*_ of *O.tauri* is large (1.01 × 10^8^). The large estimate is consistent with a previous study, which calculated an *N*_*e*_ of 1.2 ×10^7^ in *O.tauri*^50^ and provides further evidence that reduced *N*_*e*_ as a cause of genome reduction is unlikely in this representative species.

### Reduction of genome information content and DNA repair

The positive relationship between proteome size and number of DNA repair pathways is not a prediction of the BQH, but may be explained by the concept of a proteomic constraint on DNA repair^27^. The relationship has been observed in DNA viruses and prokaryotes^30^, and in picocyanobacteria^71^, but this is the first demonstration of a statistical relationship in eukaryotes, although a qualitative link between loss of DNA repair and reduced genome size has been noted in the microsporidia^72^.

The amount of information in a genome (approximated to the proteome size, *P*) is expected to be related to the mutation rate (µ) as follows^30^:

1 $$\mu =k{(2{N}_{e}\overline{s}\pi P)}^{-1}$$

where *N*_*e*_ is the effective population size, $\bar{s}$ is the average selection coefficient of a mutation (which will be deleterious on average), *π* is the genomic heterozygosity (per bp), *P* is the proteome size (in amino acids) and *k* is a proportionality constant. Both heterozygosity and selection coefficient are expected to be affected by population size: *π* is expected to be inversely related to population size^73^, while *s* is positively related to population size^74^. Hence, these two factors will have a tendency to cancel each other out, given a change in population size. This is because, while *N*_*e*_ may reduce the average selection coefficient ($\bar{s}$), it will increase the mutation load (*πP*).

The loss of genes involved in informational pathways in addition to DNA repair was observed, and may also be explained by the PCH, if such genes are involved in informational fidelity. Each step of genetic information transfer, be it replication, or gene expression, involves molecular mechanisms that maintain the fidelity of genetic information. Such fidelity-maintaining mechanisms would experience loosened selection under a reduced proteomic constraint, as the mutational load, whether the mutations are genotypic (at the level of DNA), or phenotypic (at the level of mRNA or protein^75^), would be less if the mutational target is smaller. Thus, a reduction in the number of genes involved in informational pathways is expected as proteome size reduces, as a result of the reduction in the quantity of coding information present.

## Conclusion

We have conducted a comparative genomic analysis to test the effect of Black Queen, Proteomic Constraint and genetic drift on genome reduction of PPEs. The study provides data consistent with gene loss proposed by the BQH. Further work might entail experimental measurement of the metabolites produced by some of the biosynthetic genes lost from the PPEs. When additional PPE genomes from diverse lineages are sequenced, then some convergence should be observed in the genes that are lost, if they are influenced by the metabolites present in ocean water. The BQH implies that membrane transporters should diversify in order to scavenge external metabolites, as biosynthetic genes are lost from the genome. However, an overall increase in membrane transporters was not observed as proteome size reduces in the PPEs. A potential explanation is that existing transporters fulfil this function without undergoing duplication and divergence. A range of informational genes are lost as genome size reduces, which is difficult to explain under a public goods framework. The PCH provides an explanation for the loss of informational genes, which is expected as a secondary consequence of genome reduction, as the size of the mutational target is also reduced. Furthermore, this study revealed a relatively large *N*_*e*_ for *O.tauri*, which is the smallest free-living eukaryotes yet described and a model organism for the study of biological processes in photosynthetic eukaryotes. This suggests that genetic drift (caused by reduced *N*_*e*_) as a cause of genome reduction is unlikely in PPEs.

## Supplementary information

Supplementary Material.
