# Supplementary material for: Synergism between the Black Queen effect and the proteomic constraint on genome size reduction in the photosynthetic picoeukaryotes
Source: Sci Rep. 2020 Jun 2;10:8918. doi: 10.1038/s41598-020-65476-1 (PMC7265537; doi:10.1038/s41598-020-65476-1)
Supplement: Supplementary file 1 — Supplementary Material. [file 41598_2020_65476_MOESM1_ESM.pdf]

Supplementary Material

Synergism between the Black Queen effect and the proteomic constraint on genome size reduction in the photosynthetic picoeukaryotes

Derilus, D., Rahman, M.Z., Pinero, F., Massey, S.E.

Comparison of distance measures

As an example, we consider the case of three species D, E, F and their metabolic networks on pathways a, b, c. Suppose that $D = [a, a, a, a, b, b-, b, b\ ], E = \{a, a, a, a, c, c, c, c\}$ and $F = \{a, a, a, a, b, b, c, c, c, c, \}$

For the unweighted Jaccard index we only consider whether a species contains a pathway or not. Note that D contains a and b, E contains a and c, F contains a, b and c .

If we consider $D = \{a, b\}, E = \{a, c\}, F = \{a, b, c\}$. Note that $D \cap E = \{a\}, D \cap F = \{a, b\}, E \cap F = \{a, c\}$ and $D \cup E = D \cup F = E \cup F = \{a, b, c\}$.

In this case

$$Jac(D, E) = 1 - \frac{1}{3} = \frac{2}{3} = 0.667$$

$$Jac(D, F) = 1 - \frac{2}{3} = \frac{1}{3} = 0.334$$

$$Jac(E, F) = 1 - \frac{2}{3} = \frac{1}{3} = 0.334$$

For the weighted Jaccard index, we consider species D, E, F as the following vectors:

$$D = [4,4,0], E = [4,0,4], F = [4,2,3]$$

Note the first positions describes the strength of factor a in each species, the second position describes the strength of b and the third positions describes the strength of c.

To compute the weighted Jaccard index, we add the maxima and minima and then divide.

$$WJac(D,E) = 1 - \frac{\min(4,4) + \min(4,0) + \min(0,4)}{\max(4,4) + \max(4,0) + \max(0,4)} = 1 - \frac{4}{12} = 1 - \frac{1}{3} = \frac{2}{3} = 0.667$$

$$WJac(D,F) = 1 - \frac{\min(4,4) + \min(4,2) + \min(0,3)}{\max(4,4) + \max(4,2) + \max(0,3)} = 1 - \frac{6}{11} = \frac{5}{11} = 0.455$$

$$WJac(E,F) = 1 - \frac{\min(4,4) + \min(0,2) + \min(4,3)}{\max(4,4) + \max(0,2) + \max(4,3)} = 1 - \frac{7}{10} = \frac{3}{10} = 0.30$$

To compute the Canberra distance, we first compare each entry of the vector first, and then add

$$Can(D,E) = \frac{|4-4|}{4+4} + \frac{|4-0|}{4+0} + \frac{|0-4|}{0+4} = 0 + 1 + 1 = 2$$

$$Can(D,F) = \frac{|4-4|}{4+4} + \frac{|4-2|}{4+2} + \frac{|0-3|}{0+3} = 0 + \frac{2}{6} + 1 = \frac{4}{3}$$

$$Can(E,F) = \frac{|4-4|}{4+4} + \frac{|0-2|}{0+2} + \frac{|4-3|}{4+3} = 0 + 1 + \frac{1}{7} = \frac{8}{7}$$

The Canberra distance is normalized by dividing by the number of nonzero elements.

$$AdCan(D,E) = 2\left(\frac{1}{4}\right) = 0.5$$

$$AdCan(D,F) = \frac{4}{3}\left(\frac{1}{5}\right) = \frac{4}{15} = 0.267$$

$$AdCan(E,F) = \frac{8}{7}\left(\frac{1}{5}\right) = \frac{8}{35} = 0.229$$

We may also divide by the total number of positions in the vectors, to normalize the Canberra distance.

$$Can(D,E) = \frac{2}{3} = 0.667$$

$$Can(D,F) = \frac{4}{9} = 0.364$$

$$Can(E,F) = \frac{8}{21} = 0.381$$

We propose to compute the similarity indices computed as in the Jaccard index and combine them as in the Adkins form of the Canberra distance.

If NZ is the number of nonzero positions in both x and y then the *Termwise Jaccard Index is*

$$TJac(x,y) = \frac{1}{NZ}\sum \frac{|x_i - y_i|}{\max(x_i, y_i)}$$

In our examples, then the distances become:

$$TJac(D,E) = \frac{1}{4}\left(\frac{|4-4|}{4} + \frac{|4-0|}{4} + \frac{|0-4|}{4}\right) = \frac{1}{4}(0+1+1) = \frac{2}{4} = 0.5$$

$$TJac(D,F) = \frac{1}{5}\left(\frac{|4-4|}{4} + \frac{|4-2|}{4} + \frac{|0-3|}{3}\right) = \frac{1}{5}\left(0 + \frac{2}{4} + 1\right) = \frac{3}{10} = 0.3$$

$$TJac(E,F) = \frac{1}{5}\left(\frac{|4-4|}{4} + \frac{|0-2|}{2} + \frac{|4-3|}{4}\right) = \frac{1}{5}\left(0 + 1 + \frac{1}{4}\right) = \frac{1}{4} = 0.25$$

If we perform the scaling by the number of positions in the vectors, then the distances become:

In our examples, then the distances become:

$$TJac(D,E) = \frac{1}{3}\left(\frac{|4-4|}{4} + \frac{|4-0|}{4} + \frac{|0-4|}{4}\right) = \frac{1}{3}(0+1+1) = \frac{2}{3} = 0.667$$

$$TJac(D,F) = \frac{1}{3}\left(\frac{|4-4|}{4} + \frac{|4-2|}{4} + \frac{|0-3|}{3}\right) = \frac{1}{3}\left(0 + \frac{2}{4} + 1\right) = \frac{1}{2} = 0.5$$

$$TJac(E,F) = \frac{1}{3}\left(\frac{|4-4|}{4} + \frac{|0-2|}{2} + \frac{|4-3|}{4}\right) = \frac{1}{3}\left(0 + 1 + \frac{1}{4}\right) = \frac{5}{12} = 0.417$$

Supplementary Figure 1 Efficiency of the multiple species orthogroup annotation approach (M2) compared to the single species approach (M1). The x axis presents the percentage of orthogroups that are assigned to KEGG Orthology based on the two approaches. The M2 ID mapping approach improves significantly the KEGG gene family annotation.

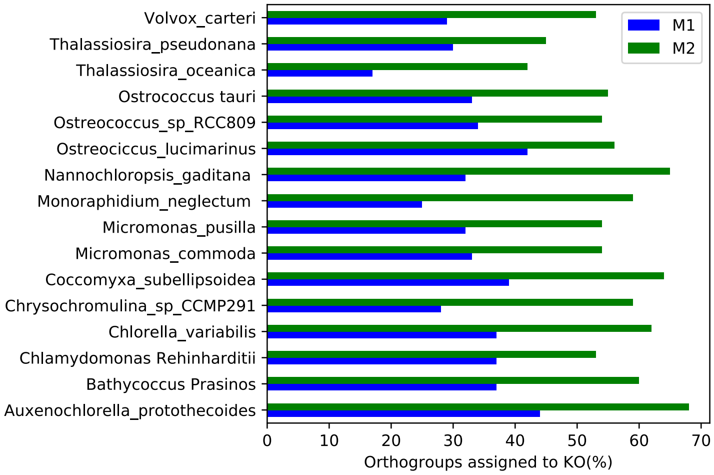

Supplementary Figure 2 Further details of the orthogroup analysis. A) A histogram of the number of assigned species to each OG. The number of OGs inferred for all the 16 species (14651 OGs) found, the size of the core OGs (554 orthogroups were found in all the algae species), species specific OGs (309 OGs were found in any one species) and the number of OGs found in any number of species. B) Scatter plot showing the number of assigned genes per OG (OG size). The average OG size was 10 genes, 4004 (27%) of the total OGs consist of only two genes. C) Histogram of the number of OGs inferred for each species, outlining the contribution of KO assigned and unassigned OGs. D) Histogram of the number of genes, highlighting the contributions from KO assigned and unassigned genes.

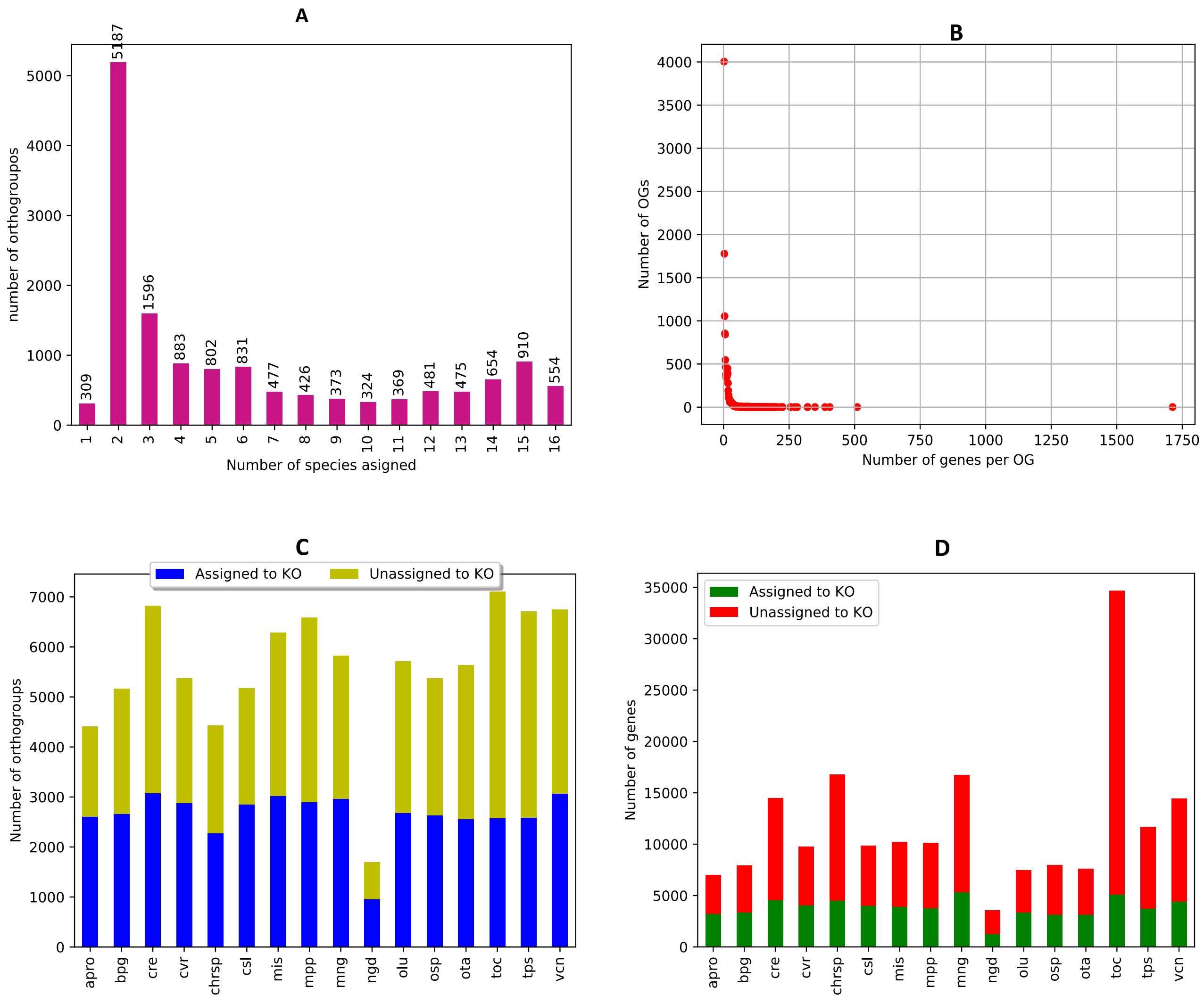

Supplementary Figure 3 (A) Boxplot showing the differences in number of orthogroups between PMA and PPEs. The blue dot outlier represents *N.gaditana*, which has the smallest number of OGs (1694) (B) Boxplot showing the differences in number of coding genes between PPEs and PMA. The red and the blue dot outliers represent the highest (34642) and the lowest (3554) number of coding genes, corresponding to *T.oceanica* and *N.gaditana* respectively.There are no significant (ns) differences in the average number of OGs (gene family) between PPEs and PMA, however significant differences were found in the number of coding genes between PPEs and PMA (t-test, p-value < 0.05).

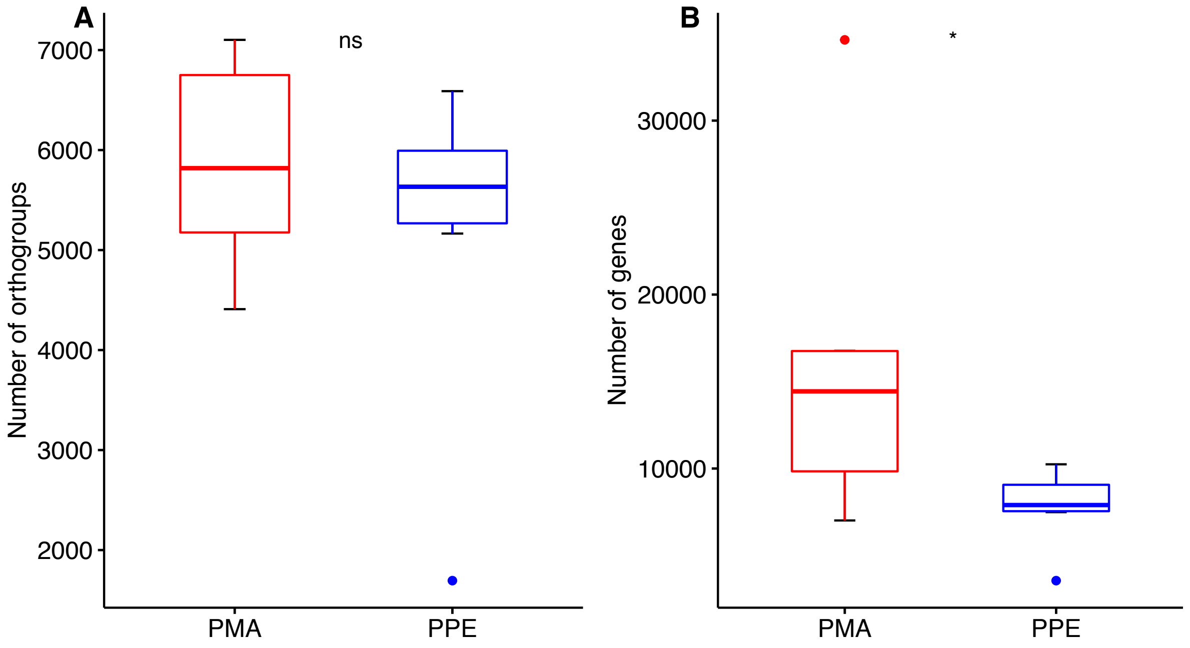

Supplementary Figure 4 Neighbor joining tree generated from unweighted (A) and weighted (B) Jaccard distance matrix of the 16 genomic metabolomic networks. The metabolomic networks of the PPE species are highlighted with an asterisk.

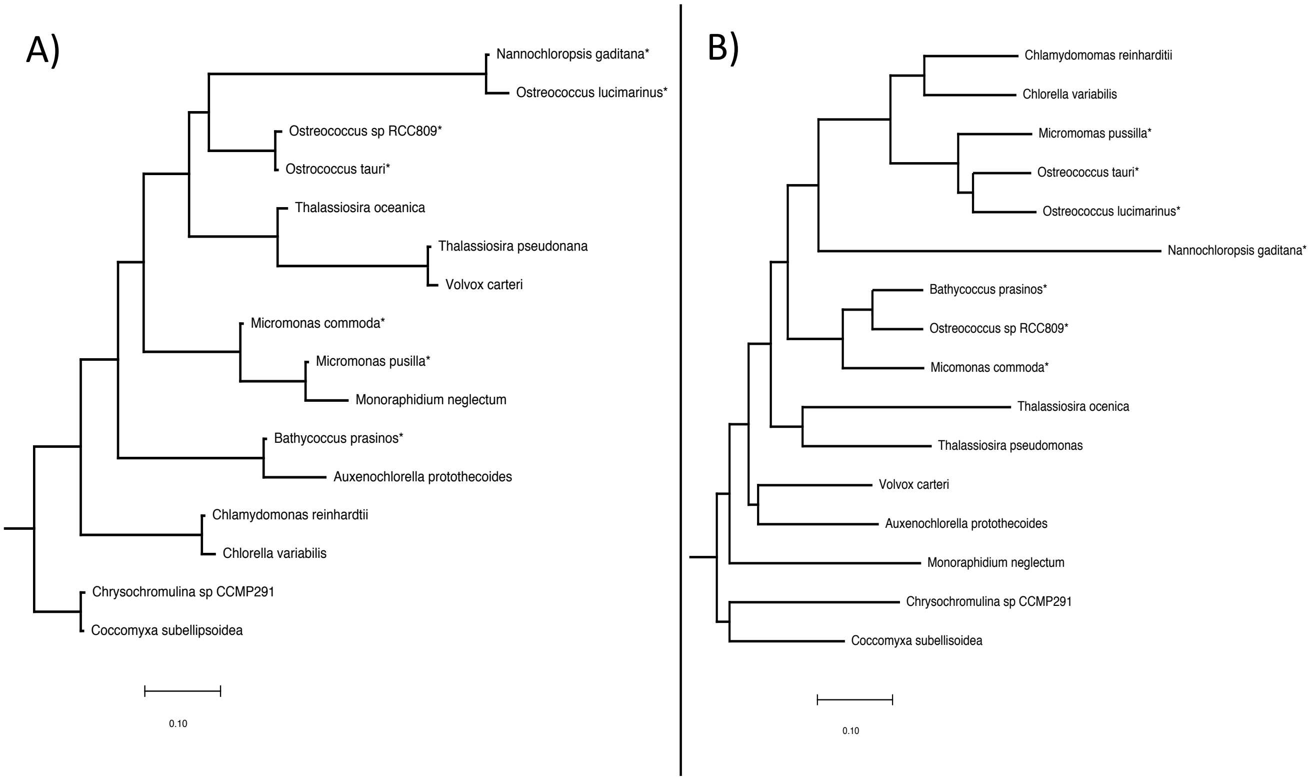

Supplementary Figure 5: The top panels show the relation of genome size (a) and proteome size (b) to the neighbor joining metabolic network tree of the 16 algae species generated using the weighted Canberra distance. The bottom panels show the genome size (c) and proteome size (d) variation in the context of the inferred phylogenomic phylogeny**.** The PPE species are highlighted with an asterisk.

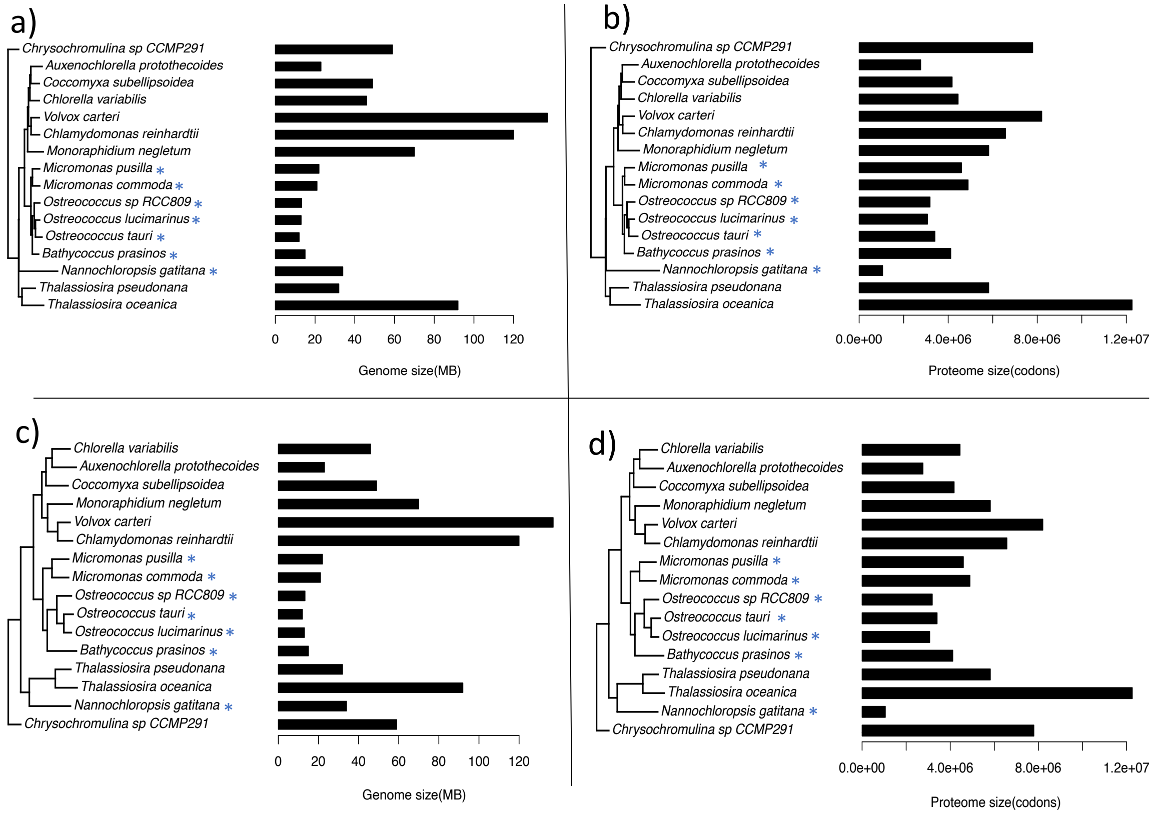

Supplementary Figure 6 Relationship between proteome size and evolutionary rate

The evolutionary rate for each species corresponds to the branch length from the phylogenomic tree, from the ancestral of the common ancestor for all species to the branch tip for each lineage.

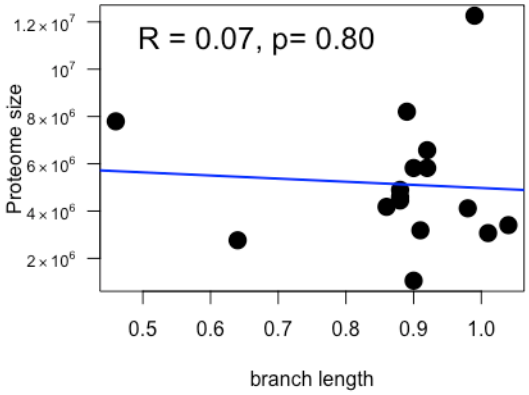

Supplementary Figure 7 MSMC estimates of effective population size with different values of expectation–maximization (EM) iterations. (A) EM iterations varied from 20 to 1000 (x-axis) and maximum likelihood estimates (y-axis). (B) MSMC effective population size estimates, using different numbers of iterations. An asymptotic effective population size and log likelihood is reached with a minimum of 100 iterations.

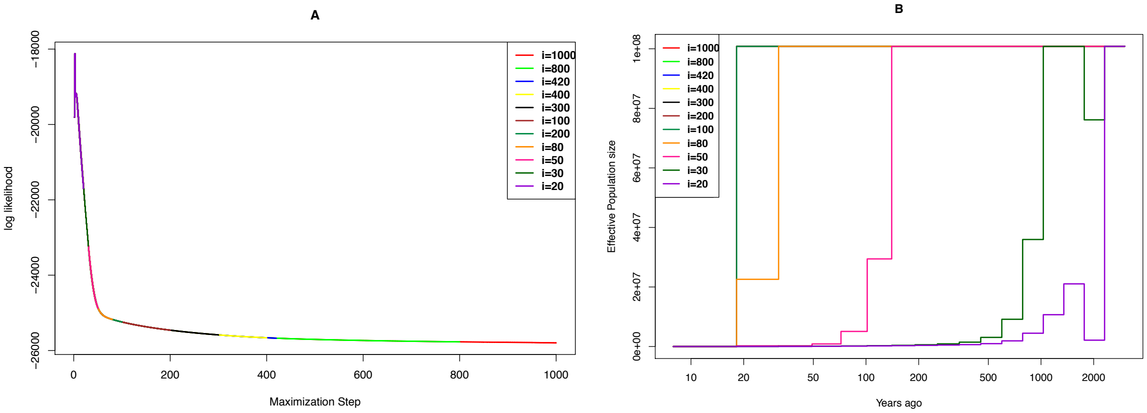

Supplementary Table 1 Table of the key results of gene family (OG) analysis for the 16 single-celled photosynthetic algae. Each species is associated with a KEGG ID. Total number of genes (genes in OGs and unassigned genes), total OGs (shared, core and specifics OGs) are presented for each species.

**Species**		**Genes**			**Orthogroups**			
**Name**	**Code**	**Total**	**In orthogroups**	**Unassigned**	**Total**	**Shared**	**Core**	**Unique**
Auxenochlorella protothecoides	apro	7014	5893	1121	4409	3846	554	5
Batycoccus Prasinos	bpg	7900	7078	822	5165	4584	554	9
Chlamydomonas reinhardtii	cre	14488	11605	2883	6817	6244	554	27
Chlorella variabilis	cvr	9780	8712	1068	5372	4778	554	19
Chrysochromulina sp_CCMP291	chrsp	16765	10648	6117	4432	3868	554	40
Coccomyxa_ Subellipsoidea	csl	9839	7960	1879	5176	4614	554	10
Micromonas pusilla	mps	10242	9154	1088	6282	5718	554	8
Micromonas commoda	mco	10137	9181	956	6589	6034	554	9
Monoraphidium neglectum	mng	16755	11159	5596	5819	5264	554	21
Nannochloropsis gaditana	ngd	3554	2477	1077	1694	1039	554	10
Ostreococcus_sp_RCC809	osp	7492	7228	264	5705	5133	554	3
Ostreococcus tauri	ota	7994	6943	1051	5370	4789	554	1
Ostreococcus lucimarinus	olu	7603	7437	166	5633	5076	554	1
Thalassiosira oceanica	toc	34642	18126	16516	7102	6539	554	101
Thalassiosira pseudonana	tps	11673	10159	1514	6704	6145	554	18
Volvox carteri	vcn	14436	11403	3033	6750	6175	554	27

Supplementary Table 2 Significant positive correlations between KEGG pathways and proteome size. The correlation coefficient (R) and the statistical significance (p-value) are given for all the molecular function that exhibit significant positive correlation with proteome size. After PIC correction, we observed that a total of 83 molecular functions showed a significant positive correlation with proteome size ($R \leq 0.5$, p-value ≤ 0.05). From these we have identified 44 strong ($R \leq 0.7$), and 39 moderate ($0.5 < R < 0.7$) positive correlations.

Third level KEGG pathway	R	p-value	Range in number of genes	Second level KEGG pathway
Glycine@ serine and threonine metabolism [PATH:ko00260]	0.75	0.001	4-20	Amino acid metabolism
Lysine biosynthesis [PATH:ko00300]	0.71	0.002	1-8	
Lysine degradation [PATH:ko00310]	0.70	0.002	1-8	
Alanine@ aspartate and glutamate metabolism [PATH:ko00250]	0.67	0.004	4-12	
Arginine biosynthesis [PATH:ko00220]	0.62	0.011	2-14	
Cysteine and methionine metabolism [PATH:ko00270]	0.61	0.012	5-25	
Histidine metabolism [PATH:ko00340]	0.56	0.023	1-14	
Phenylalanine@ tyrosine and tryptophan biosynthesis [PATH:ko00400]	0.53	0.034	2-17	
Tyrosine metabolism [PATH:ko00350]	0.53	0.035	0-4	
Valine@ leucine and isoleucine biosynthesis [PATH:ko00290]	0.50	0.047	3-12	
Monobactam biosynthesis [PATH:ko00261]	0.52	0.041	1-10	Biosynthesis of other secondary metabolites
Chemical carcinogenesis [PATH:ko05204]	0.56	0.025	2-13	Cancers: Overview
Choline metabolism in cancer [PATH:ko05231]	0.53	0.033	0-3	
Pentose and glucuronate interconversions [PATH:ko00040]	0.90	0.000	0-6	Carbohydrate metabolism
Pentose phosphate pathway [PATH:ko00030]	0.73	0.000	0-13	
Glyoxylate and dicarboxylate metabolism [PATH:ko00630]	0.73	0.001	1-13	
Amino sugar and nucleotide sugar metabolism [PATH:ko00520]	0.70	0.000	13-65	
Glycolysis / Gluconeogenesis [PATH:ko00010]	0.63	0.009	1-6	
Fat digestion and absorption [PATH:ko04975]	0.66	0.005	0-2	Digestive system
Bile secretion [PATH:ko04976]	0.51	0.202	0-1	
Antifolate resistance [PATH:ko01523]	0.77	0.000	1-9	Drug resistance: Antineoplastic
Non-alcoholic fatty liver disease (NAFLD) [PATH:ko04932]	0.72	0.002	8-47	Endocrine and metabolic diseases
Thyroid hormone synthesis [PATH:ko04918]	0.63	0.009	2-12	Endocrine system

Methane metabolism [PATH:ko00680]	0.59	0.020	1-6	Energy metabolism
Carbon fixation in photosynthetic organisms [PATH:ko00710]	0.53	0.034	3-21	
Plant-pathogen interaction [PATH:ko04626]	0.64	0.010	0-10	Environmental adaptation
Sulfur relay system [PATH:ko04122]	0.84	0.000	0-10	Folding@ sorting and degradation
RIG-I-like receptor signaling pathway [PATH:ko04622]	0.83	0.000	0-3	Immune system
Human papillomavirus infection [PATH:ko05165]	0.63	0.009	0-19	Infectious diseases: Viral
Influenza A [PATH:ko05164]	0.57	0.020	0-2	
Secondary bile acid biosynthesis [PATH:ko00121]	0.86	0.000	0-3	
Arachidonic acid metabolism [PATH:ko00590]	0.83	0.000	0-2	
Sphingolipid metabolism [PATH:ko00600]	0.67	0.004	0-7	Lipid metabolism
Fatty acid degradation [PATH:ko00071]	0.55	0.027	0-2	
Nicotinate and nicotinamide metabolism [PATH:ko00760]	0.81	0.000	7-29	
One carbon pool by folate [PATH:ko00670]	0.68	0.004	3-13	
Riboflavin metabolism [PATH:ko00740]	0.65	0.006	2-10	Metabolism of cofactors
**Third level KEGG pathway**	**R**	**p-value**	**Range in number of genes**	**Second level KEGG pathway**
Ubiquinone and other terpenoid-quinone biosynthesis [PATH:ko00130]	0.63	0.009	3-20	and vitamins
Pantothenate and CoA biosynthesis [PATH:ko00770]	0.49	0.054	3-20	
Taurine and hypotaurine metabolism [PATH:ko00430]	0.79	0.000	0-4	Metabolism of
beta-Alanine metabolism [PATH:ko00410]	0.50	0.049	0-11	other amino acids
Monoterpenoid biosynthesis [PATH:ko00902]	0.80	0.000	0-2	Metabolism of terpenoids
Carotenoid biosynthesis [PATH:ko00906]	0.75	0.001	7-17	and polyketides
Biosynthesis of ansamycins [PATH:ko01051]	0.57	0.020	0-2	
Huntington disease [PATH:ko05016]	0.55	0.027	3-18	Neurodegenerative diseases
Function unknown	0.70	0.003	4-42	Poorly characterized
Ubiquitin system [BR:ko04121]	0.89	0.000	41-260	
Chromosome and associated proteins [BR:ko03036]	0.89	0.000	100-465	
Membrane trafficking [BR:ko04131]	0.79	0.000	58-252	
Translation factors [BR:ko03012]	0.77	0.001	19-98	
DNA repair and recombination proteins [BR:ko03400]	0.77	0.001	58-246	
Mitochondrial biogenesis [BR:ko03029]	0.72	0.002	57-196	Protein families:
Spliceosome [BR:ko03041]	0.71	0.002	56-219	genetic information processing
Transfer RNA biogenesis [BR:ko03016]	0.69	0.003	48-149	
Messenger RNA biogenesis [BR:ko03019]	0.66	0.005	26-111	
Proteasome [BR:ko03051]	0.63	0.009	15-51	
Chaperones and folding catalysts [BR:ko03110]	0.63	0.010	14-96	
Ribosome biogenesis [BR:ko03009]	0.61	0.013	67-226	
Ribosome [BR:ko03011]	0.60	0.014	36-191	
Glycosyltransferases [BR:ko01003]	0.74	0.000	14-97	

Peptidases [BR:ko01002]	0.74	0.001	26-125	Protein families: metabolism
Photosynthesis proteins [BR:ko00194]	0.63	0.009	5-82	
Protein kinases [BR:ko01001]	0.61	0.010	16-88	
Protein phosphatases and associated proteins [BR:ko01009]	0.55	0.027	13-60	
Lipid biosynthesis proteins [BR:ko01004]	0.55	0.029	19-54	
Amino acid related enzymes [BR:ko01007]	0.51	0.043	9-34	
Exosome [BR:ko04147]	0.79	0.000	50-273	Protein families: signaling and cellular processes
Transporters [BR:ko02000]	0.68	0.004	75-333	
G protein-coupled receptors [BR:ko04030]	0.65	0.007	0-5	
Proteoglycans [BR:ko00535]	0.63	0.009	0-2	
Cytoskeleton proteins [BR:ko04812]	0.59	0.017	18-145	
GTP-binding proteins [BR:ko04031]	0.52	0.039	3-27	
Pattern recognition receptors [BR:ko04054]	0.51	0.045	1-148	
Plant hormone signal transduction [PATH:ko04075]	0.57	0.020	1-9	Signal transduction
Two-component system [PATH:ko02020]	0.55	0.027	0-5	
AMPK signaling pathway [PATH:ko04152]	0.49	0.053	0-6	
Alcoholism [PATH:ko05034]	0.71	0.000	0-4	Substance dependence
mRNA surveillance pathway [PATH:ko03015]	0.83	0.000	0-2	Translation
Aminoacyl-tRNA biosynthesis [PATH:ko00970]	0.65	0.007	0-5	
Peroxisome [PATH:ko04146]	0.71	0.002	12-59	Transport and catabolism
Transcription	0.98	0.980	1-30	Unclassified: genetic information processing
Protein processing	0.66	0.000	2-9	
Energy metabolism	0.67	0.004	0-7	Unclassified: metabolism
Enzymes with EC numbers	0.65	0.007	44-129	
Signaling proteins	0.62	0.010	2-28	Unclassified: signaling and cellular processes
Toluene degradation [PATH:ko00623]	0.73	0.001	0-5	Xenobiotics biodegradation and metabolism
Drug metabolism - other enzymes [PATH:ko00983]	0.65	0.007	1-18	
Styrene degradation [PATH:ko00643]	0.51	0.044	0-6	

Supplementary Table 3 KEGG molecular functions that did not exhibit significant positive correlations with proteome size (R < 0.5 and p > 0.05) at 95% confidence intervals.

Third level KEGG pathway	R	p-value	Range in number of genes	Second level KEGG pathway
Longevity regulating pathway - worm [PATH:ko04212]	0.10	0.70	0-8	Aging
Longevity regulating pathway - multiple species [PATH:ko04213]	0.01	0.98	0-4	Aging
Arginine and proline metabolism [PATH:ko00330]	0.45	0.08	3-24	Amino acid metabolism
Valine@ leucine and isoleucine degradation [PATH:ko00280]	0.19	0.48	1-13	
Tryptophan metabolism [PATH:ko00380]	0.17	0.53	2-12	
Flavonoid biosynthesis [PATH:ko00941]	0.44	0.09	0-1	Biosynthesis of other secondary metabolites
Streptomycin biosynthesis [PATH:ko00521]	0.31	0.24	1-4	
Betalain biosynthesis [PATH:ko00965]	0.18	0.51	0-1	
Phenylpropanoid biosynthesis [PATH:ko00940]	0.18	0.52	1-18	
Caffeine metabolism [PATH:ko00232]	0.17	0.53	0-1	
Neomycin@ kanamycin and gentamicin biosynthesis [PATH:ko00524]	0.10	0.73	0-1	
Acarbose and validamycin biosynthesis [PATH:ko00525]	0.08	0.76	0-1	
Tropane@ piperidine and pyridine alkaloid biosynthesis [PATH:ko00960]	0.07	0.81	2-8	
Carbapenem biosynthesis [PATH:ko00332]	0.02	0.94	0-3	
Central carbon metabolism in cancer [PATH:ko05230]	0.29	0.28	0-6	Cancers: Overview
Viral carcinogenesis [PATH:ko05203]	0.06	0.84	1-10	
Proteoglycans in cancer [PATH:ko05205]	0.05	0.85	0-2	
Transcriptional misregulation in cancer [PATH:ko05202]	0.01	0.98	0-1	
Thyroid cancer [PATH:ko05216]	0.25	0.36	0-1	Cancers: Specific types
Small cell lung cancer [PATH:ko05222]	0.25	0.36	1-2	
Renal cell carcinoma [PATH:ko05211]	0.18	0.51	1-9	
Non-small cell lung cancer [PATH:ko05223]	0.15	0.58	0-1	
Hepatocellular carcinoma [PATH:ko05225]	0.04	0.87	0-1	
Propanoate metabolism [PATH:ko00640]	0.32	0.23	0-2	Carbohydrate metabolism
Fructose and mannose metabolism [PATH:ko000051]	0.21	0.44	1-20	
Pyruvate metabolism [PATH:ko00620]	0.20	0.45	8-14	
Butanoate metabolism [PATH:ko00650]	0.19	0.47	1-3	
Inositol phosphate metabolism [PATH:ko00562]	0.18	0.51	0-5	
Ascorbate and aldarate metabolism [PATH:ko00053]	0.17	0.52	4-14	
Galactose metabolism [PATH:ko00052]	0.14	0.60	1-7	

Citrate cycle (TCA cycle) [PATH:ko00020]	0.13	0.62	0-3	
Starch and sucrose metabolism [PATH:ko00500]	0.11	0.69	1-35	
Fluid shear stress and atherosclerosis [PATH:ko05418]	0.09	0.75	0-1	Cardiovascular diseases
Cellular senescence [PATH:ko04218]	0.40	0.12	0-1	
Apoptosis - fly [PATH:ko04214]	0.38	0.14	0-2	
Cell cycle - yeast [PATH:ko04111]	0.36	0.17	1-2	Cell growth and death
p53 signaling pathway [PATH:ko04115]	0.29	0.27	0-5	
Necroptosis [PATH:ko04217]	0.04	0.89	0-1	
Regulation of actin cytoskeleton [PATH:ko04810]	0.06	0.83	0-1	Cell motility
Adherens junction [PATH:ko04520]	0.24	0.36	0-2	
Tight junction [PATH:ko04530]	0.01	0.97	0-1	
Quorum sensing [PATH:ko02024]	0.30	0.26	1-4	Cellular community - prokaryotes
Biofilm formation - Vibrio cholerae [PATH:ko05111]	0.19	0.49	0-4	
Biofilm formation - Pseudomonas aeruginosa [PATH:ko02025]	0.08	0.77	1-3	
Vitamin digestion and absorption [PATH:ko04977]	0.45	0.08	0-3	
Salivary secretion [PATH:ko04970]	0.32	0.23	0-1	
Protein digestion and absorption [PATH:ko04974]	0.27	0.30	0-2	Digestive system
**Third level KEGG pathway**	**R**	**p-value**	**Range in number of genes**	**Second level KEGG pathway**
Mineral absorption [PATH:ko04978]	0.24	0.36	0-4	
Cholesterol metabolism [PATH:ko04979]	0.11	0.67	0-14	
Carbohydrate digestion and absorption [PATH:ko04973]	0.05	0.86	0-3	
beta-Lactam resistance [PATH:ko01501]	0.04	0.87	0-1	
Platinum drug resistance [PATH:ko01524]	0.29	0.28	0-2	Drug resistance: Antineoplastic
Endocrine resistance [PATH:ko01522]	0.01	0.97	0-2	
Type I diabetes mellitus [PATH:ko04940]	0.17	0.52	0-4	
Insulin resistance [PATH:ko04931]	0.08	0.77	0-13	Endocrine and
Cushing syndrome [PATH:ko04934]	0.01	0.97	0-2	metabolic diseases
Glucagon signaling pathway [PATH:ko04922]	0.46	0.07	0-5	
PPAR signaling pathway [PATH:ko03320]	0.20	0.45	3-13	Endocrine system
Parathyroid hormone synthesis@ secretion and action [PATH:ko04928]	0.18	0.51	0-3	
Melanogenesis [PATH:ko04916]	0.14	0.60	0-1	
Sulfur metabolism [PATH:ko00920]	0.29	0.28	1-7	
Nitrogen metabolism [PATH:ko00910]	0.22	0.41	2-12	
Oxidative phosphorylation [PATH:ko00190]	0.20	0.45	4-12	Energy metabolism
Carbon fixation pathways in prokaryotes [PATH:ko00720]	0.01	0.96	3-10	

Thermogenesis [PATH:ko04714]	0.27	0.32	0-3	Environmental adaptation
Proximal tubule bicarbonate reclamation [PATH:ko04964]	0.08	0.77	0-3	Excretory system
Protein processing in endoplasmic reticulum [PATH:ko04141]	0.48	0.06	4-23	Folding@ sorting
Protein export [PATH:ko03060]	0.30	0.26	1-5	and degradation
Other glycan degradation [PATH:ko00511]	0.40	0.13	0-10	
Glycosylphosphatidylinositol (GPI)-anchor biosynthesis [PATH:ko00563]	0.27	0.31	1-15	Glycan biosynthesis and metabolism
Glycosphingolipid biosynthesis - globo and isoglobo series [PATH:ko00603]	0.09	0.73	0-11	
N-Glycan biosynthesis [PATH:ko00510]	0.09	0.74	1-5	
Systemic lupus erythematosus [PATH:ko05322]	0.30	0.27	0-5	
Rheumatoid arthritis [PATH:ko05323]	0.20	0.45	0-8	
Primary immunodeficiency [PATH:ko05340]	0.11	0.70	0-3	Immune diseases
NOD-like receptor signaling pathway [PATH:ko04621]	0.33	0.21	0-1	
Antigen processing and presentation [PATH:ko04612]	0.02	0.95	0-4	
Vibrio cholerae infection [PATH:ko05110]	0.31	0.24	0-4	
Tuberculosis [PATH:ko05152]	0.26	0.33	0-3	Infectious diseases: Bacterial
Bacterial invasion of epithelial cells [PATH:ko05100]	0.10	0.70	0-2	
African trypanosomiasis [PATH:ko05143]	0.37	0.15	0-2	Infectious diseases: Parasitic
Amoebiasis [PATH:ko05146]	0.28	0.30	0-1	
Hepatitis B [PATH:ko05161]	0.40	0.13	0-1	Infectious diseases: Viral
Human T-cell leukemia virus 1 infection [PATH:ko05166]	0.34	0.19	0-4	
Steroid hormone biosynthesis [PATH:ko00140]	0.48	0.06	0-3	
Glycerophospholipid metabolism [PATH:ko00564]	0.45	0.08	8-29	
alpha-Linolenic acid metabolism [PATH:ko00592]	0.44	0.09	1-16	
Glycerolipid metabolism [PATH:ko00561]	0.21	0.43	0-7	
Ether lipid metabolism [PATH:ko00565]	0.20	0.45	0-13	Lipid metabolism
Synthesis and degradation of ketone bodies [PATH:ko00072]	0.16	0.55	0-4	
Cutin@ suberine and wax biosynthesis [PATH:ko00073]	0.11	0.70	0-3	
Steroid biosynthesis [PATH:ko00100]	0.10	0.70	2-11	
Fatty acid elongation [PATH:ko00062]	0.07	0.78	0-30	
Fatty acid biosynthesis [PATH:ko00061]	0.06	0.83	0-6	
Primary bile acid biosynthesis [PATH:ko00120]	0.01	0.98	0-1	
Porphyrin and chlorophyll metabolism [PATH:ko00860]	0.46	0.07	9-54	
Vitamin B6 metabolism [PATH:ko00750]	0.40	0.10	4-9	
Thiamine metabolism [PATH:ko00730]	0.35	0.19	1-9	
**Third level KEGG pathway**	**R**	**p-value**	**Range in number of genes**	**Second level KEGG pathway**

Retinol metabolism [PATH:ko00830]<br>Lipoic acid metabolism [PATH:ko00785]<br>Folate biosynthesis [PATH:ko00790]<br>Biotin metabolism [PATH:ko00780]	0.27<br>0.18<br>0.14<br>0.08	0.30<br>0.51<br>0.60<br>0.78	0-3<br>2-4<br>6-17<br>0-5	Metabolism of cofactors
Cyanoamino acid metabolism [PATH:ko00460]<br>Glutathione metabolism [PATH:ko00480]<br>Selenocompound metabolism [PATH:ko00450]<br>Phosphonate and phosphinate metabolism [PATH:ko00440]	0.45<br>0.35<br>0.30<br>0.04	0.08<br>0.19<br>0.26<br>0.87	0-5<br>3-17<br>2-7<br>0-5	Metabolism of other amino acids
Terpenoid backbone biosynthesis [PATH:ko00900]<br>Sesquiterpenoid and triterpenoid biosynthesis [PATH:ko00909]	0.39<br>0.05	0.13<br>0.85	5-27<br>0-1	Metabolism of terpenoids and polyketides
Retrograde endocannabinoid signaling [PATH:ko04723]<br>GABAergic synapse [PATH:ko04727]	0.29<br>0.09	0.27<br>0.73	0-2<br>0-3	Nervous system
Amyotrophic lateral sclerosis (ALS) [PATH:ko05014]<br>Prion diseases [PATH:ko05020]<br>Alzheimer disease [PATH:ko05010]	0.26<br>0.21<br>0.03	0.34<br>0.43<br>0.92	0-4<br>0-3<br>1-4	Neurodegenerative diseases
Pyrimidine metabolism [PATH:ko00240]<br>Purine metabolism [PATH:ko00230]	0.48<br>0.41	0.06<br>0.12	3-15<br>10-27	Nucleotide metabolism
General function prediction only	0.32	0.22	3-13	Poorly characterized
DNA replication proteins [BR:ko03032]<br>Transcription machinery [BR:ko03021]<br>Transcription factors [BR:ko03000]	0.43<br>0.19<br>0.08	0.10<br>0.48<br>0.76	10-42<br>14-104<br>7-104	Protein families: genetic information processing
Prenyltransferases [BR:ko01006]<br>Peptidoglycan biosynthesis and degradation proteins [BR:ko01011]<br>Lipopolysaccharide biosynthesis proteins [BR:ko01005]<br>Polyketide biosynthesis proteins [BR:ko01008]<br>Cytochrome P450 [BR:ko00199]	0.38<br>0.19<br>0.18<br>0.15<br>0.03	0.14<br>0.48<br>0.50<br>0.59<br>0.91	2-17<br>0-2<br>0-7<br>0-1<br>2-14	Protein families: metabolism
Glycosaminoglycan binding proteins [BR:ko00536]<br>Secretion system [BR:ko02044]<br>Ion channels [BR:ko04040]<br>Prokaryotic defense system [BR:ko02048]<br>Lectins [BR:ko04091]<br>CD molecules [BR:ko04090]<br>Glycosylphosphatidylinositol (GPI)-anchored proteins [BR:ko00537]	0.45<br>0.41<br>0.39<br>0.32<br>0.25<br>0.19<br>0.07	0.08<br>0.11<br>0.13<br>0.22<br>0.34<br>0.48<br>0.80	1-63<br>12-48<br>3-21<br>0-18<br>3-11<br>0-5<br>0-3	Protein families: signaling and cellular processes

Phototransduction - fly [PATH:ko04745]	0.11	0.68	0-8	Sensory system
MAPK signaling pathway - yeast [PATH:ko04011]	0.46	0.07	1-5	Signal transduction
Phosphatidylinositol signaling system [PATH:ko04070]	0.36	0.17	8-18	
Sphingolipid signaling pathway [PATH:ko04071]	0.28	0.29	3-14	
mTOR signaling pathway [PATH:ko04150]	0.26	0.32	1-5	
MAPK signaling pathway - plant [PATH:ko04016]	0.19	0.48	0-8	
Ras signaling pathway [PATH:ko04014]	0.04	0.87	0-1	
Wnt signaling pathway [PATH:ko04310]	0.02	0.94	1-8	
Morphine addiction [PATH:ko05032]	0.22	0.42	0-27	Substance dependence
RNA transport [PATH:ko03013]	0.34	0.20	0-3	Translation
Autophagy - yeast [PATH:ko04138]	0.48	0.06	0-1	Transport and catabolism
Lysosome [PATH:ko04142]	0.27	0.30	0-23	
Autophagy - animal [PATH:ko04140]	0.18	0.51	0-2	
Endocytosis [PATH:ko04144]	0.06	0.83	0-2	
Translation	0.46	0.07	2-10	Unclassified: genetic information processing
Cofactor metabolism	0.47	0.07	0-4	
**Third level KEGG pathway**	**R**	**p-value**	**Range in number of genes**	**Second level KEGG pathway**
Lipid metabolism	0.20	0.45	1-4	Unclassified: metabolism
Carbohydrate metabolism	0.04	0.88	0-2	
Amino acid metabolism	0.03	0.91	2-8	
Others	0.37	0.16	0-6	Unclassified: signaling and cellular processes
Cell growth	0.37	0.16	0-2	
Structural proteins	0.35	0.18	2-15	
Cell motility	0.28	0.29	0-11	
Transport	0.06	0.84	4-18	
Drug metabolism - cytochrome P450 [PATH:ko00982]	0.31	0.24	0-8	Xenobiotics biodegradation and metabolism
Chloroalkane and chloroalkene degradation [PATH:ko00625]	0.25	0.36	0-1	
Polycyclic aromatic hydrocarbon degradation [PATH:ko00624]	0.13	0.64	0-3	
Metabolism of xenobiotics by cytochrome P450 [PATH:ko00980]	0.11	0.68	0-1	
Atrazine degradation [PATH:ko00791]	0.07	0.79	0-1	
Aminobenzoate degradation [PATH:ko00627]	0.04	0.90	0-1	

Supplementary Table 4 Correlation between genes involved in biogenesis and biosynthesis pathways, with proteome size. From a total of 249 KEGG catalogued pathways, 40 (16%) of them are identified as biogenesis and biosynthesis pathways. The degree of reduction was calculated from the change in gene numbers from the largest proteome to the smallest proteome, using the function corresponding to the best-fit line.

Third-level KEGG pathway	Correlation coefficient (R)	p-value	Degree of reduction (%)	Second level KEGG pathway
Arginine biosynthesis [PATH:ko00220]	0.61	0.012	62	Amino acid metabolism
Lysine biosynthesis [PATH:ko00300]	0.71	0.002	86	
Phenylalanine@ tyrosine and tryptophan biosynthesis [PATH:ko00400]	0.51	0.042	41	
Valine@ leucine and isoleucine biosynthesis [PATH:ko00290]	0.49	0.051	44	
Carbapenem biosynthesis [PATH:ko00332]	0.02	0.928	38	Biosynthesis of other secondary metabolites
Monobactam biosynthesis [PATH:ko00261]	0.49	0.052	39	
Phenylpropanoid biosynthesis [PATH:ko00940]	0.2	0.467	53	
Streptomycin biosynthesis [PATH:ko00521]	0.39	0.136	60	
Tropane@ piperidine and pyridine alkaloid biosynthesis [PATH:ko00960]	0.03	0.906	7	
Chemical carcinogenesis [PATH:ko05204]	0.61	0.013	77	Cancers: Overview
Viral carcinogenesis [PATH:ko05203]	0.04	0.895	1	
Glycolysis / Gluconeogenesis [PATH:ko00010]	0.57	0.020	38	Carbohydrate metabolism
Parathyroid hormone synthesis@ secretion and action [PATH:ko04928]	0.24	0.379	69	Endocrine system
Thyroid hormone synthesis [PATH:ko04918]	0.69	0.003	71	
Melanogenesis [PATH:ko04916]	0.13	0.627	81	
Thermogenesis [PATH:ko04714]	0.3	0.251	118	Environmental adaptation
Glycosphingolipid biosynthesis - globo and isoglobo series [PATH:ko00603]	0.09	0.752	63	Glycan biosynthesis and metabolism
Glycosylphosphatidylinositol (GPI)-anchor biosynthesis [PATH:ko00563]	0.23	0.384	91	
N-Glycan biosynthesis [PATH:ko00510]	0.13	0.630	34	
Cutin@ suberine and wax biosynthesis [PATH:ko00073]	0.48	0.063	62	

Fatty acid biosynthesis [PATH:ko00061]	0.07	0.808	45	Lipid metabolism
Steroid biosynthesis [PATH:ko00100]	0.12	0.655	21	
Steroid hormone biosynthesis [PATH:ko00140]	0.47	0.064	55	
Secondary bile acid biosynthesis [PATH:ko00121]	0.86	0.000	91	
Folate biosynthesis [PATH:ko00790]	0.13	0.645	15	Metabolism of cofactors and vitamins
Pantothenate and CoA biosynthesis [PATH:ko00770]	0.42	0.102	23	
Ubiquinone and other terpenoid-quinone biosynthesis [PATH:ko00130]	0.6	0.015	45	
Terpenoid backbone biosynthesis [PATH:ko00900]	0.41	0.118	48	Metabolism of cofactors o terpenoids and polyketides
Biosynthesis of ansamycins [PATH:ko01051]	0.59	0.020	73	
Carotenoid biosynthesis [PATH:ko00906]	0.75	0.001	59	
Monoterpenoid biosynthesis [PATH:ko00902]	0.79	0.000	130	
Messenger RNA biogenesis [BR:ko03019]	0.62	0.010	37	Protein families: genetic information processing
Ribosome biogenesis [BR:ko03009]	0.6	0.013	32	
Transfer RNA biogenesis [BR:ko03016]	0.69	0.003	38	
Mitochondrial biogenesis [BR:ko03029]	0.73	0.001	47	
Lipid biosynthesis proteins [BR:ko01004]	0.54	0.030	40	Protein families: metabolism
Lipopolysaccharide biosynthesis proteins [BR:ko01005]	0.23	0.394	82	
Peptidoglycan biosynthesis and degradation proteins [BR:ko01011]	0.17	0.524	54	
Photosynthesis proteins [BR:ko00194]	0.64	0.008	62	
Aminoacyl-tRNA biosynthesis [PATH:ko00970]	0.63	0.360	61	Translation
